# Groundwater–surface water interactions and resilience to hydrological extremes in a data-sparse arid transboundary river basin: Insights from collaborative groundwater modelling

**DOI:** 10.1007/s10040-026-03143-x

**Published:** 2026-08-14

**Authors:** Syed Md Touhidul Mustafa, Jean-Christophe Comte, Fulvio Franchi, Alessia Matano, Anne F. Van Loon, Sithabile Tirivarombo, Luis Artur, Zareen Bharucha, Annatoria Chinyama, Farisse Chirindja, Rosie Day, Joyce Dube, Stephen Hussey, Edward Nesamvuni, Wandile Nomquphu, Oluwaseun Olabode, Melanie Rohse, Simon Taylor, Jarmo de Vries

**Affiliations:** 1 School of Geosciences, University of Aberdeen, Aberdeen, UK; 2 Now at Hydrology and Environmental Hydraulics Group, Wageningen University & Research, Wageningen, the Netherlands; 3 Dipartimento di Scienze della Terra e Geoambientali, Università degli studi di Bari – Aldo Moro, Bari, Italy; 4 Institute for Environmental Studies, Vrije Universiteit Amsterdam, Amsterdam, The Netherlands; 5 Department of Earth and Environmental Science, Botswana International University of Science and Technology (BIUST), Palapye, Botswana; 6 Eduardo Mondlane University, Maputo, Mozambique; 7 Anglia Ruskin University, Cambridge, UK; 8 National University of Science and Technology, Bulawayo, Zimbabwe; 9 School of Geography, Earth and Environmental Sciences, University of Birmingham, University of Birmingham, BirminghamBirmingham, UK; 10 Dabane Trust, Bulawayo, Zimbabwe; 11 Department of Sustainable Food Systems and Development, University of Free State, Bloemfontein, South Africa; 12 Water Research Commission, Pretoria, South Africa

**Keywords:** Managed aquifer recharge (MAR), Groundwater/surface water relations, Hydrological extremes, Transboundary stakeholder engagement, Limpopo River Basin (LRB)

## Abstract

Sustainable groundwater management is key to building resilience to hydrological extremes but remains challenging in data-scarce transboundary river basins. In this study, a transboundary, collaborative, physically based groundwater modelling approach has been developed for the Limpopo River Basin (LRB), which is highly vulnerable to floods and droughts. Monitoring data and model results consistently suggest that prolonged wet periods and floods significantly raised groundwater levels, whereas prolonged droughts decreased them, particularly in the upstream and central regions of the basin characterized by low-storage fractured bedrock aquifers. Groundwater–surface water interactions are greater downstream, where water tables are shallower in unconsolidated superficial materials, and intensify during floods, especially in the central region. Modelling results also suggest that baseflow is less responsive to extremes than groundwater recharge across the LRB, highlighting the groundwater system’s attenuation capacity. The model was subsequently used to assess and discuss a series of co-created management scenarios focused specifically on improving groundwater storage during wet periods for use during dry periods. They included: (1) managed aquifer recharge (MAR), using (1a) injection well(s) next to large dams, (1b) rainwater and runoff harvesting (RRH) and infiltration through local wells, (1c) RRH through local ponds and (1d) small reservoirs (e.g. sand dams); (2) deforestation; (3) afforestation; and (4) increased groundwater abstraction. Scenarios suggest that strategy (1b), when widely applied across the basin, may be the most effective to reduce the risk and impact of floods, droughts, and groundwater depletion by supporting the maintenance of groundwater levels and baseflow, while reducing storm runoff.

**Supplementary Information:**

The online version contains supplementary material available at https://doi.org/10.1007/s10040-026-03143-x.

## Résumé

La gestion durable des eaux souterraines est essentielle pour renforcer la résilience face aux extrêmes hydrologiques, mais elle demeure complexe dans les bassins hydrographiques transfrontaliers où les données sont rares. Dans cette étude, une approche de modélisation collaborative, transfrontalière et fondée sur des processus physiques a été développée pour le bassin du fleuve Limpopo (LRB), une zone très vulnérable aux inondations et aux sécheresses. Les données de suivi et les résultats du modèle s’accordent à indiquer que les périodes humides prolongées et les inondations ont entraîné une hausse significative des niveaux des nappes d’eaux souterraines, tandis que les sécheresses prolongées les ont fait baisser, en particulier dans les régions amont et centrale du bassin, caractérisées par des aquifères en socle fracturé à faible capacité de stockage. Les interactions entre eaux souterraines et eaux de surface sont plus marquées dans les régions aval, où les nappes sont plus superficielles au sein de formations meubles, et s'intensifient lors des crues, notamment dans la région centrale. Les résultats de la modélisation suggèrent également que le débit de base réagit moins aux événements extrêmes que la recharge des nappes à l'échelle du bassin, soulignant ainsi la capacité d'atténuation du système aquifère. Le modèle a ensuite servi à évaluer et à discuter une série de scénarios de gestion élaborés conjointement, axés spécifiquement sur l'amélioration du stockage des eaux souterraines durant les périodes humides en vue de leur utilisation lors des périodes sèches. Ces scénarios comprenaient : (1) la recharge gérée des aquifères (MAR), via (1a) des puits d'injection à proximité de grands barrages, (1b) la collecte des eaux de pluie et de ruissellement (RRH) avec infiltration par des puits locaux, (1c) la RRH via des mares locales, et (1d) de petits réservoirs (p. ex. : barrages de sable) ; (2) la déforestation ; (3) le (re)boisement ; et (4) l'augmentation des prélèvements d'eau souterraine. Les scénarios suggèrent que la stratégie (1b), si elle est appliquée à grande échelle sur l'ensemble du bassin, pourrait être la plus efficace pour réduire les risques et les impacts des inondations, des sécheresses et de l'épuisement des nappes, en favorisant le maintien des niveaux piézométriques et du débit de base tout en réduisant le ruissellement lié aux précipitations intenses.

## Resumen

La gestión sostenible de las aguas subterráneas es clave para construir resiliencia ante extremos hidrológicos, pero sigue siendo un reto en cuencas fluviales transfronterizas con pocos datos. En este estudio, se ha desarrollado un enfoque transfronterizo, colaborativo y basado en la física de modelización de aguas subterráneas para la Cuenca del Río Limpopo (LRB), que es altamente vulnerable a inundaciones y sequías. Los datos de monitoreo y los resultados de los modelos sugieren consistentemente que los períodos húmedos prolongados y las inundaciones elevaron significativamente los niveles de agua subterránea, mientras que las sequías prolongadas los redujeron, especialmente en las regiones aguas arriba y central de la cuenca caracterizadas por acuíferos rocosos fracturados de bajo almacenamiento. Las interacciones entre agua subterránea y agua superficial son mayores aguas abajo, donde los niveles freáticos son más superficiales en materiales superficiales no consolidados, y se intensifican durante las inundaciones, especialmente en la región central. Los resultados de la modelización también sugieren que el flujo base responde menos a los extremos que la recarga de agua subterránea a lo largo del LRB, lo que destaca la capacidad de atenuación del sistema de aguas subterráneas. El modelo se utilizó posteriormente para evaluar y debatir una serie de escenarios de gestión co-creados centrados específicamente en mejorar el almacenamiento de agua subterránea durante periodos húmedos para su uso en periodos secos. Incluían: (1) recarga gestionada del acuífero (MAR), utilizando (1a) pozo(s) de inyección(es) junto a grandes presas, (1b) captación de agua de lluvia y escorrentía (RRH) e infiltración a través de pozos locales, (1c) RRH a través de estanques locales, y (1d) pequeños embalses (por ejemplo, presas de arena); (2) deforestación; (3) reforestación; y (4) aumentar la captación de agua subterránea. Los escenarios sugieren que la estrategia (1b), cuando se aplica ampliamente en toda la cuenca, puede ser la más eficaz para reducir el riesgo e impacto de inundaciones, sequías y agotamiento de las aguas subterráneas, apoyando el mantenimiento de los niveles de agua subterránea y el caudal base, al tiempo que se reduce la escorrentía pluvial.

## Abstract (zh)

可持续地下水管理是提升流域对水文极端事件韧性的关键, 但在数据稀缺的跨界河流流域中, 这一目标仍面临较大挑战。本研究针对极易遭受洪水和干旱影响的Limpopo河流域 (Limpopo River Basin, 简称LRB) , 构建了一种跨界协同、基于物理过程的地下水模拟方法。监测数据与模拟结果一致表明, 持续湿润期及洪水事件会显著抬升地下水位, 而长期干旱则导致地下水位下降, 尤以上游和中部地区最为明显; 这些区域以低储水性的裂隙基岩含水层为主。下游地区由于松散表层沉积物中地下水埋深较浅, 地下水-地表水相互作用更为强烈, 并且在洪水期间进一步增强, 尤其是在中部地区。模拟结果还表明, 与地下水补给相比, 基流对极端事件的响应在整个LRB中较弱, 显示出地下水系统的缓冲能力。随后, 研究基于共同构建的管理情景, 围绕如何在湿润期提高地下水储量、并在干旱期加以利用, 对一系列方案进行了评估与讨论。具体包括: (1) 含水层人工补给 (managed aquifer recharge , 简称MAR) , 涵盖 (1a) 在大型水坝附近设置注入井, (1b) 通过当地井进行雨水和径流集蓄 (rainwater and runoff harvesting , 简称RRH) 及入渗补给, (1c) 通过当地池塘实施RRH, 以及 (1d) 建设小型蓄水设施 (如沙坝) ; (2) 毁林; (3) 造林; (4) 增加地下水开采。情景分析表明, 若在全流域广泛实施策略 (1b) , 其在维持地下水位和基流、减少暴雨径流的同时, 可能是降低洪水、干旱及地下水枯竭风险与影响的最有效措施。

## Resumo

A gestão sustentável das águas subterrâneas é fundamental para construir resiliência à extremos hidrológicos, mas continua desafiadora em bacias hidrográficas transfronteiriças com poucos dados. Neste estudo, foi desenvolvida uma abordagem transfronteiriça, colaborativa e baseada fisicamente em modelagem de águas subterrâneas para a Bacia do Rio Limpopo (BRL), que é altamente vulnerável a inundações e secas. Dados de monitoramento e resultados de modelos sugerem consistentemente que períodos úmidos prolongados e inundações elevaram significativamente os níveis de água subterrânea, enquanto secas prolongadas os diminuíram, especialmente nas regiões a montante e central da bacia caracterizadas por aquíferos rochosos fraturados de baixo armazenamento. As interações entre água subterrânea e água superficial são maiores rio abaixo, onde os lençóis freáticos são mais rasos em materiais superficiais não consolidados, e se intensificam durante enchentes, especialmente na região central. Os resultados da modelagem também sugerem que o fluxo de base responde menos a extremos do que a recarga de água subterrânea em toda a BRL, destacando a capacidade de atenuação do sistema de água subterrânea. O modelo foi posteriormente utilizado para avaliar e discutir uma série de cenários de manejo cocriados, focados especificamente na melhoria do armazenamento de água subterrânea durante períodos úmidos para uso durante períodos secos. Incluíam: (1) recarga de aquífero gerenciada (RAG), usando (1a) poços de injeção próximos a grandes barragens, (1b) captação de água da chuva e escoamento (CACE) e infiltração por poços locais, (1c) CACE através de lagoas locais e (1d) pequenos reservatórios (por exemplo, barragens de areia); (2) desmatamento; (3) reflorestamento; e (4) aumento da captação de água subterrânea. Cenários sugerem que a estratégia (1b), quando amplamente aplicada em toda a bacia, pode ser a mais eficaz para reduzir o risco e o impacto de enchentes, secas e esgotamento da água subterrânea, apoiando a manutenção dos níveis das águas subterrâneas e do fluxo de base, ao mesmo tempo em que reduz o escoamento das chuvas.

## Introduction

The frequency and severity of extreme hydrological events are increasing in most regions of the world as a consequence of climate change (AghaKouchak et al. 2015; IPCC 2021; Raymond et al. 2022). These changes are also projected for, and already manifesting in, southern Africa which has been referred to as a climate change hotspot (WMO 2024). The Limpopo River Basin (LRB) in southern Africa is one of the world’s most exposed and vulnerable to floods and droughts (Kusangaya et al. 2014; Thornton et al. 2014). Several studies have reported an increase in the frequency and intensity of extreme hydrological events in the LRB in the past few decades (Spaliviero et al. 2014; Trambauer et al. 2015; and Botai et al. 2020). Recurrent extreme events and their observed increase in magnitude and frequency pose a serious threat to water and food security. Population growth and associated anthropogenic activities and infrastructures impose additional pressures on food and water security and could worsen the impact on water availability and quality by exacerbating the frequency and severity of hydrological extreme events (IPCC 2021).

Transboundary aquifers (TBAs) are vital for drinking water, agriculture, natural ecosystems, and millions of livelihoods worldwide (Davies et al. 2013; Wada and Heinrich 2013). However, they have been largely overlooked compared to transboundary river basins (TRBs) (Puri and Aureli 2005). With approximately 468 TBAs across 142 countries, this highlights their global significance and the urgent need for coordinated management (IGRAC 2021). Despite the recognised importance of understanding and managing transboundary freshwater resources for conflict prevention, sustainable management, and cooperation (Best 2019), 60% of TRBs lack proper cooperation frameworks (UNTC 2019). When it comes to TBAs, the situation is even worse, as the majority lack formal cooperative frameworks—highlighting the need for improved knowledge-based management and tools (e.g. models) for informed decision-making (UNILC 2008; Bakker and Duncan 2017). However, such tools and management recommendations are limited in both TRBs and TBAs due to data scarcity and a lack of groundwater-relevant cooperative frameworks.

While large-scale (continental to global) studies have shed light on hydrological extremes and their effects on water resources, as well as the relationship between climate factors and hydrological processes (Veldkamp et al. 2018; de Graaf et al. 2019), local studies have concentrated on specific regions, addressing localised concerns, and emphasising the significance of context-specific factors in water management (Goderniaux et al. 2011, Mustafa et al. 2019, Najafi et al. 2023). In contrast, there has been limited research conducted at intermediate scales, e.g. large river basins, which can serve as a crucial link between broad-scale processes, local data, and social issues, ultimately guiding the implementation of effective and fair water management practices.

Floods and droughts are often studied and managed separately. However, the impacts of floods can be influenced by prior droughts and vice versa (Quesada-Montano et al. 2018; Mustafa et al. 2021; Franchi et al. 2024; Barendrecht et al. 2024). Additionally, hydrological extremes and their management in one region/country may have significant consequences in neighbouring regions/countries in a TRB such as the LRB.

As global water demand increases and surface water availability becomes less reliable under an increasingly variable climate, groundwater, which is the primary water source for many people (including more than 70% of people in the LRB; Pietersen and Beekman 2016), is being increasingly relied upon, playing an essential role in building resilience to hydrological extremes. However, the potential for groundwater to be safely and sustainably used and managed across large transboundary basins is poorly understood, and groundwater over-abstraction in parts of a large basin (e.g. upstream) may have detrimental effects on groundwater and surface water availability in other parts (e.g. downstream) (Kuang et al. 2024).

Identification of appropriate water, including groundwater, governance strategies is important (i) to ensure sustainable water resources management and (ii) to improve climate adaptation and resilience to hydrological extremes. Nevertheless, integrated water resources management is complex and requires balancing different interests of different stakeholders (Voinov and Gaddis 2008; Sterling et al. 2019; Voinov et al. 2018). The participation of stakeholders in (i) identifying appropriate management strategies, (ii) water resources planning, and (iii) decision-making processes, is essential for an effective and inclusive integrated water resources management (IWRM) approach to ensure sustainable water management (GWP 2000; Voinov and Gaddis 2008; Elshall et al. 2020). Accordingly, the approaches to water resources management have undergone significant transformations from a largely prevailing top-down, sector-specific managerial and planning approach to more IWRM (GWP 2000), including bottom-up, user-driven multi-disciplinary contributions. However, identification of sustainable water management and adaption strategies is usually dealt with separately by one type of stakeholder rather than co-created, although co-creation is increasingly recognised as essential (Dias et al. 2020).

Hydrological models offer flexible platforms for co-creation and knowledge exchange that are increasingly used to (i) understand and forecast the effects of hydrological systems under anthropogenic and climatic influences, (ii) evaluate alternative management scenarios to provide information for improved decision-making, and (iii) to engage a diverse range of stakeholders in the co-design of management strategies, including researchers, policymakers, practitioners, and users (Basco-Carrera et al. 2017; Jones et al. 2020; Rangecroft et al. 2018; Van Loon et al. 2025). Nevertheless, the predictive reliability of such models is not only influenced by the hydrological and computational uncertainties (Ajami et al. 2007; Refsgaard et al. 2007; Mustafa et al. 2020) but also by uncertainties arising from the stakeholders’ and modellers’ qualitative conceptual inputs and perspectives. Therefore, it is essential to integrate local people’s knowledge, management approach, and stakeholders’ social, political, and economic perspectives in the modelling framework (Herman et al. 2019; Mustafa et al. 2021; Rangecroft et al. 2018).

It is widely accepted that broad stakeholder participation is essential for effective water governance, enhancing the credibility, legitimacy, and reliability of water management decisions. Engaging stakeholders in the decision-making process promotes transparency, social learning, and consensus building, leading to more sustainable and inclusive water management (White et al. 2010; Heink et al. 2015; Basco-Carrera et al. 2017; Loucks and Van Beek 2017). Various groundwater management regulations, including the EU Water Framework Directive (Jorgensen et al. 2017), the Australian water reform agenda (Tan et al. 2012), the California Sustainable Groundwater Management Act (Kiparsky 2016, Babbitt et al. 2018), and South Africa Water Reform (Seward 2010), among numerous others, emphasise the importance of stakeholder involvement as a key policy component. Recent real-world examples also further highlight the critical importance of stakeholder participation in groundwater sustainability (Griffioen et al. 2014; Leduc et al. 2017; Custodio et al. 2019). Knuppe and Pahl-Wostl (2011) identified conflicts due to excluding local stakeholders in aquifer governance in Spain. Custodio et al. (2019) showed better outcomes with stakeholder consensus in sustainable aquifer management.

While much research has focused on engaging diverse stakeholders in planning and decision-making processes, limited research has been conducted on the co-creation of hydrological models and further definition and model testing of management scenarios in collaboration with stakeholders (Melsen et al. 2018; Basco-Carrera et al. 2017). Therefore, researchers introduced participatory modelling approaches aimed at enhancing stakeholder ownership of Decision Support Tools (DSTs) by actively involving stakeholders in the modelling process. There have been various participatory modelling approaches used worldwide, with some referring to them as "participatory modelling" and others as "collaborative modelling" (Basco-Carrera et al. 2017). Wurl et al. (2018) and Perdikaki et al. (2022) both demonstrated that participatory modelling is an effective tool for assessing aquifer system resilience, engaging stakeholders, and developing sustainable water management solutions.

Despite the existence of best practice guidelines for improving the reliability, accuracy, and effectiveness of models as decision-support tools and participatory modelling approaches (Schuwirth et al. 2019; Herman et al. 2019; Badham et al. 2019), models can face significant challenges when they are used for environmental decision-support processes (Jones at al., 2020). This could be because of (i) the limited stakeholder involvement in developing the modelling framework, building the model, and evaluating its outputs, which often leads to model not being sufficiently realistic or fit-for-purpose, and (ii) the incompleteness of existing modelling guidelines, which mainly focus on the technical aspects of modelling from a modellers’ perspectives and do not include guidelines for stakeholder inputs and interactions (Basco-Carrera et al. 2017; Badham et al. 2019; Jones at al., 2020).

Additionally, in TBAs, data scarcity and highly variable data coverage and quality across TRBs’ countries and subbasins, along with the absence of robust cooperative frameworks, make it even more challenging to develop spatially distributed groundwater management tools (e.g. models) that support informed decision-making. As a result, the development and application of such tools remain highly limited, despite their critical importance for the sustainable management of TBAs. Although Leduc et al. (2017) highlighted that active communication between local stakeholders, water authorities, and researchers appears to be the sole approach to prevent or alleviate serious threats for transboundary aquifers, there is very limited research on the application of participatory or collaborative modelling approaches for groundwater management in data-scarce transboundary basins such as the LRB.

In this study, a collaborative approach has been applied in the LRB that incorporates stakeholder knowledge as an additional source of data in hydrological modelling and ensured effective stakeholder involvement iteratively throughout the entire modelling cycle, from model conception to results analysis. As part of the proposed framework, stakeholders of different levels and sectors were involved in the development of the integrated hydrological model through a suite of iterative regional and transboundary workshops which ultimately led to the collaborative identification and evaluation of drought-flood management scenarios. The objectives of this research were: (i) to evaluate the applicability and capability of the proposed collaborative modelling approach in improving the hydrogeological understanding and parameterisation of a complex, data-scarce transboundary hydrogeological model; (ii) to better understand the spatio-temporal hydrological connectivity in the LRB, including groundwater flow dynamics, groundwater–surface water (GW-SW) interactions, upstream–downstream connections, and the role of groundwater in increasing resilience to hydrological extremes; (iii) to identify and co-create management scenarios to enhance resilience to hydrological extremes and promote groundwater sustainability; and (iv) to evaluate the impact of the identified management scenarios on improving resilience and sustainability in a data-scarce transboundary basin.

## Materials and methods

### Study area

**Fig. 1:**
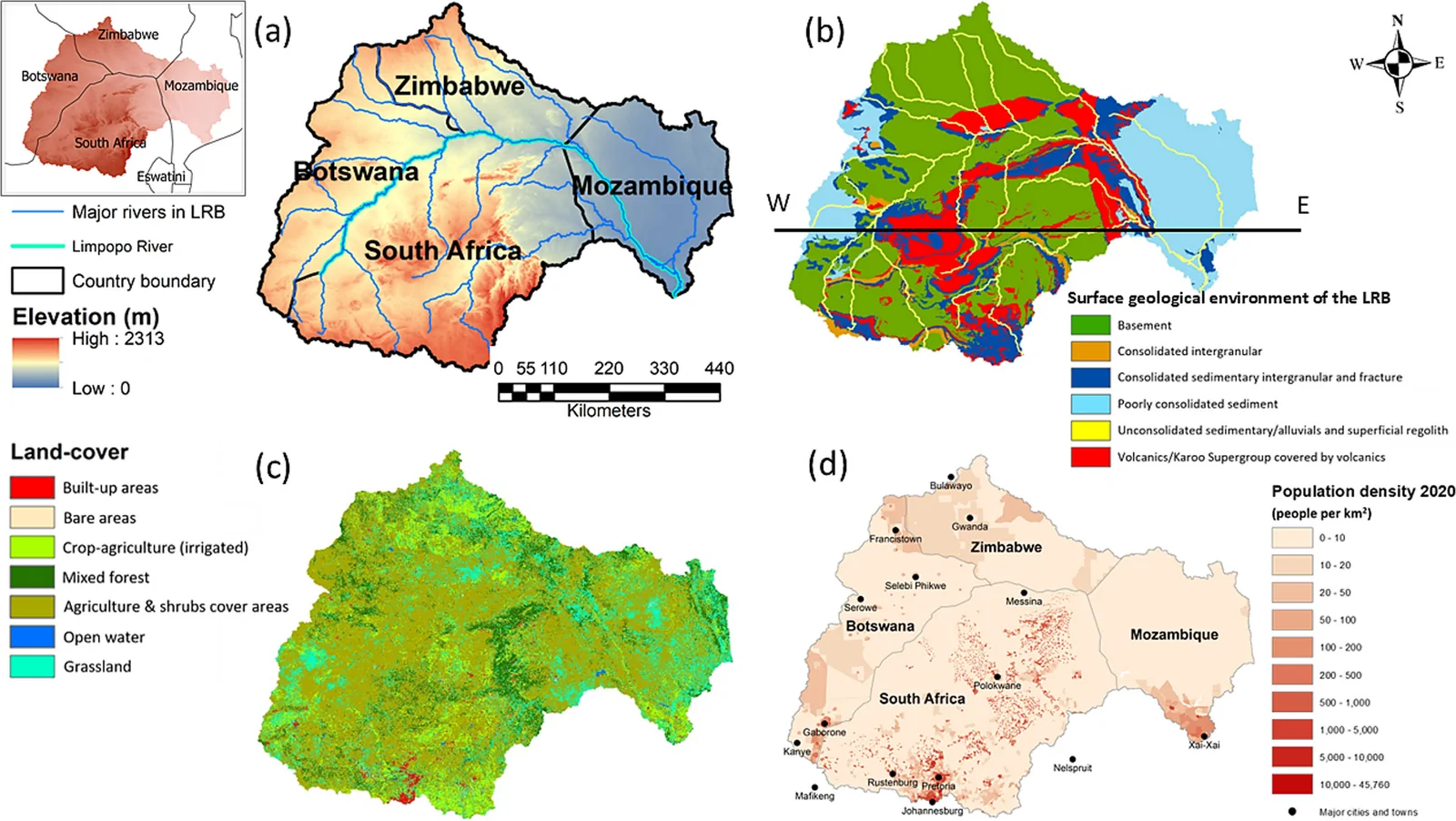
Limpopo River Basin (LRB): (a) elevation of the basin, including the Limpopo River and its major tributaries (inset: location of the basin within Africa), (b) simplified surface geology (adapted and modified from CSIR 2003; MacDonald et al. 2005, 2008; and BGS 2019). The geological cross-section along the W-E line is presented in Fig. 3, (c) land use and land cover, and (d) population density. Please refer to Table S2 of the ESM for the full sources of maps

The LRB is highly vulnerable to recurrent hydrological extremes (floods and droughts). The basin is the fourth largest transboundary river basin in southern Africa covering 415,000 km^2^, spanning across the countries of Botswana, Mozambique, South Africa, and Zimbabwe (Fig. 1a). The basin elevation ranges from sea level in the east (Mozambique) to 2313 m in the southwest (South Africa) (Fig. 1a). The Limpopo River is about 1750 km long, starting in South Africa and flowing northwards then eastwards across Botswana and Zimbabwe, terminating in the Indian Ocean in Mozambique. It marks around 613 km of the international borders between these four counties (Fig. 1a). Agricultural land and shrub cover are the dominant land cover in the basin, constituting around 71% of the total area, including rainfed or irrigated agriculture and shrub cover (Fig. 1c). Agriculture is the largest water user (70 %) with agricultural areas being more dominant in the upstream countries (Fig. 1c). The LRB is home to over 18 million people, with two national capitals (Pretoria and Gaborone) and several major cities (e.g. Johannesburg) within the basin (Fig. 1d). Approximately 83% of the total population of the basin lives in the South African part of the basin, while 7, 6, and 4% live in Botswana, Zimbabwe, and Mozambique parts, respectively (Fig. 1d). From a country’s total population perspective, 69% of Botswana’s population lives in the LRB, while 22, 10, and 7% of South Africa, Zimbabwe, and Mozambique’s populations live in the LRB, respectively (Kahinda et al. 2016).

Rainfall is not uniformly distributed, and it increases downstream. Average annual rainfall varies between 270 and 1502 mm across the basin with most rainfall occurring from October to March. Evapotranspiration (ET) is greater than rainfall throughout the year except for November, December and January.

### Multisector collaborative groundwater modelling approach

**Fig. 2:**
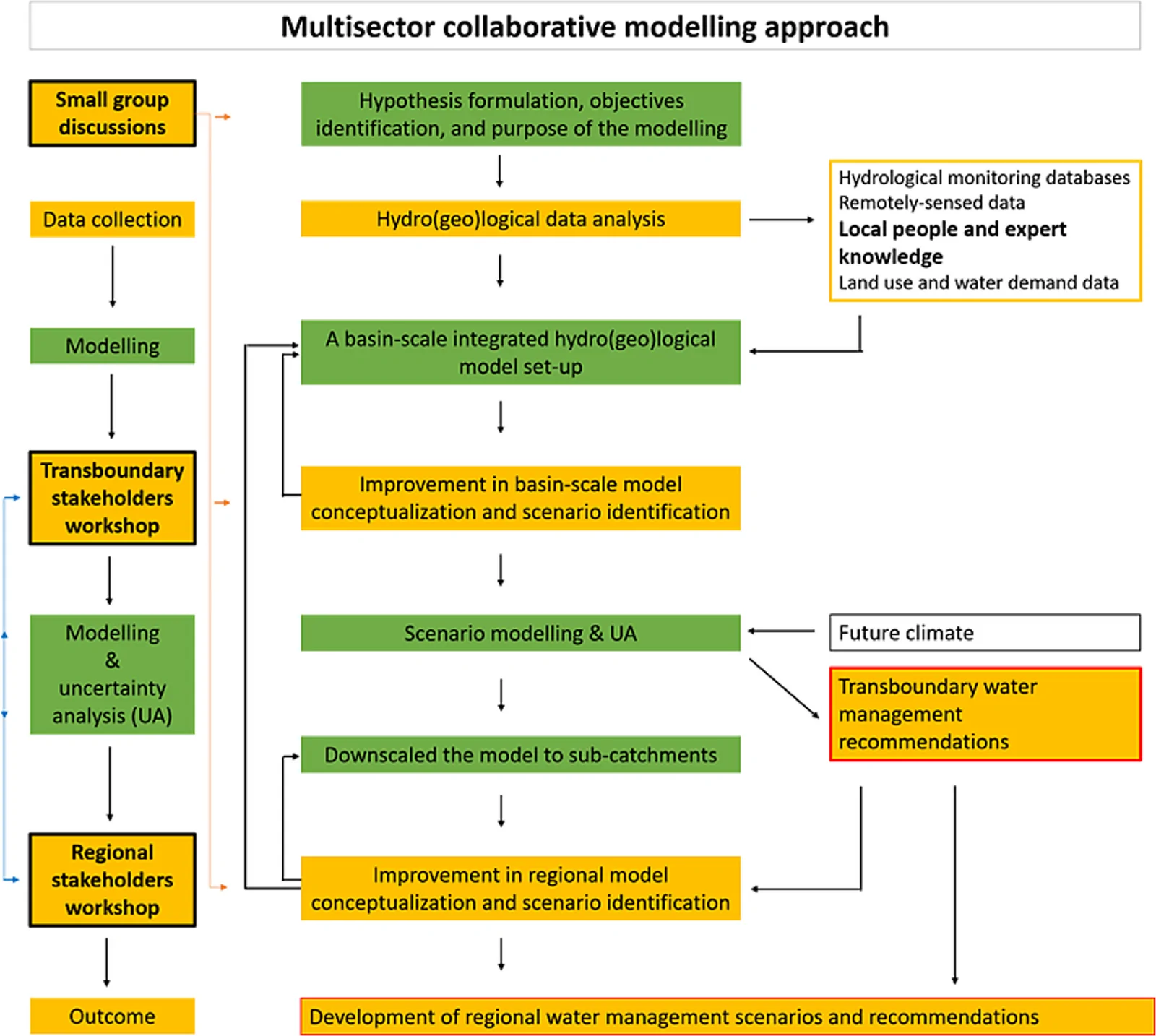
The proposed multisector collaborative modelling approach, where yellow represents the direct or indirect involvement of stakeholders. The left columns represent the activities/steps, with their respective purpose in the middle column

In this study, a multisector collaborative groundwater modelling (MCGM) approach is introduced on the basis of the application to increase resilience to hydrological extremes in the LRB, which involved stakeholders at every modelling step, starting from the model conceptualisation, through the identification, co-creation, and analysis of feasible management scenarios. This ensured effective, iterative stakeholder involvement throughout the study and considered stakeholder knowledge as an additional source of data. Expert and local people’s knowledge was incorporated iteratively in the hydrogeological model through small group discussions and regional to transboundary stakeholder workshops (Fig. 2 and Table S1 of the electronic supplementary material (ESM)). The first step of the process was to identify the key hypotheses, objectives, and purpose of the modelling exercise. The next step was to collect spatiotemporal-vertical data, including available climatic and hydrogeological data, as well as local people’s and expert knowledge, to build the model. Then through two consecutive group discussions, the modelling approach and initial model conceptualisation were discussed, and preliminary management scenarios were identified. Next, a basin-scale integrated hydrogeological model was set up using the existing datasets and according to the hypotheses and objectives that were identified in the first step, and capable of addressing all identified scenarios. The model conceptualisation, primary list of management scenarios, and preliminary results were presented and discussed in the transboundary workshops. By accommodating their suggestions and comments, the model was subsequently improved through these transboundary stakeholder workshops, and management scenarios were co-developed and explored. Subsequently, transboundary water management recommendations were produced for decision-making based on the results of the model runs. The results of the transboundary stakeholders’ workshop were then discussed during a regional workshop to further improve the model conceptualisation and develop realistic water management strategies for the region, taking into account the input of regional stakeholders. Several small group discussions were also conducted throughout the modelling study to better understand the system, improve the model, and refine the management scenarios. Finally, a set of regional and transboundary water management recommendations was produced. For more details on stakeholder workshops and stakeholder input integration, see Van Loon et al. (2025).

### Model setup under data-limiting conditions

For a conceptual hydro(geo)logical understanding of the LRB, data were compiled from open-access data servers, including hydrological monitoring databases and remote sensing data portals (Table S2 of the ESM). The major challenges of using the available data were (i) very limited availability of in situ hydrological measurement and (ii) inconsistent spatial and temporal resolutions of both in situ measurements and remote sensing data.

An integrated basin-scale hydrogeological model has been developed to achieve the objectives of the study. In this integrated model, the widely used groundwater flow model MODFLOW-2005 (Harbaugh 2005), was coupled with a spatially distributed water balance model, WetSpass (Batelaan and De Smedt 2007; Abdollahi et al. 2017), using the FloPy package in Python (Bakker et al. 2016). A steady-state model was set up as an initial step, representing the conditions of the year 2009, which was chosen as the best documented period, with most data available. This initial steady-state model was set up to improve the understanding of the hydrogeological system and to get a first estimate of the hydrogeological properties of the different geological regions. Then a transient model was set up for the period 2009–2019 based on the optimised parameters of the steady-state model and considering the simulated heads of the 2009 steady-state model as initial heads for the first stress period of the transient model.

#### Geological environment of the LRB

**Fig. 3:**
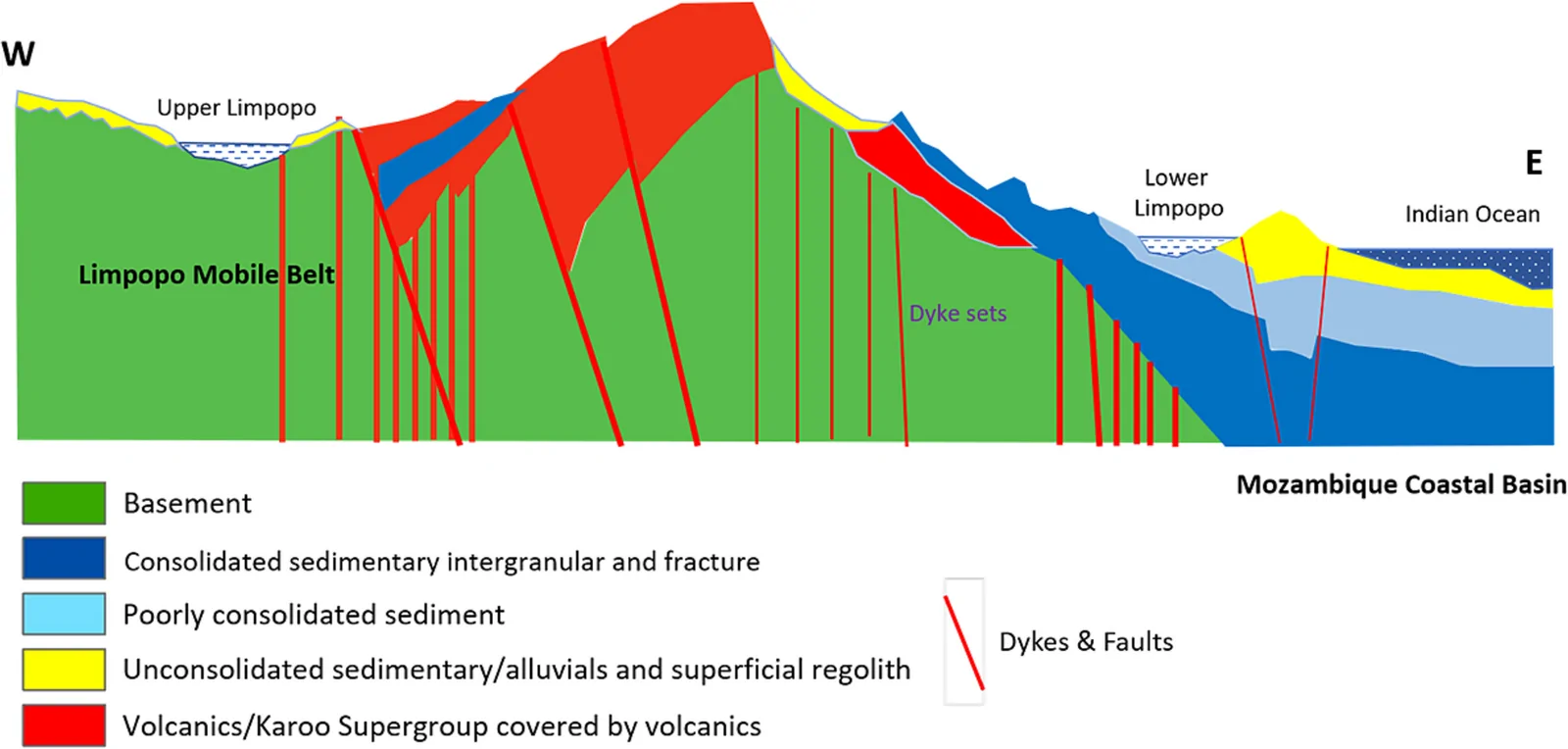
Schematic geological cross-section (W-E: Fig. 1b) of the Limpopo River Basin (modified from CSIR 2003; MacDonald et al. 2005, 2008, and BGS 2019)

The LRB groundwater model was structured according to its three main geological regions (Figs. 1b, 3).

*The Precambrian basement*: These areas are dominated by the crystalline basement complex made of hard or weathered-fractured basement rocks. The top layer in these areas typically comprises unconsolidated alluvial deposits in valleys. Below this, the second and third layers consist of two aquifers: the upper weathered basement aquifer (i.e. the saprolite), and the lower fractured basement aquifer (i.e. the saprock). The saprock is generally known as greater yielding than the saprolite (Jolly 1986; Holland 2011, 2012; Ebrahim et al. 2019; Abiye et al. 2020);

*Consolidated volcanic and sedimentary bedrock:* Covered by younger volcanic and sedimentary rocks: the top layer in these areas represents unconsolidated alluvial deposits; the middle layer represents the volcanic-sedimentary aquifer, typically composed of consolidated porous, fractured or karstified dolomite, porous/fractured sandstone, and in some areas capped by the porous/fractured volcanic rocks of the Karoo Supergroup. The bottom layer reflects the fractured basement (CSIR 2003, BGS 2019);

*The Mozambique sedimentary basin:* the top layer in these areas (lower reaches of the LRB) reflects the unconsolidated alluvial deposits, the middle layer reflects poorly consolidated sediment, and the bottom layer reflects the consolidated porous/fractured sedimentary rocks (BGS 2019).

The exposed thickness of unconsolidated alluvial deposits and superficial regolith varies between 2 and 15 m along the major river valleys, while across the LRB the upper weathered zone (the saprolite) typically ranges from 12 to 50 m in thickness, and the fractured aquifer extends to depths that can exceed 120 m (Jolly 1986; Herbert et al. 1997; CSIR 2003; Vegter, 2003; Holland 2011; Mpala et al. 2016; Walker et al. 2018; Ebrahim et al. 2019; Abiye et al. 2020; Saveca et al. 2022).

In addition to the three geological regions mentioned above, the LRB is also characterised by the occurrence of major regional faults and dykes (Fig. 3 and Fig. S2a of the ESM). These features were reported to have greater conductivities than the surrounding rocks (Dippenaar 2008; Abiye et al. 2020), leading to increased groundwater flow and vulnerability to groundwater contamination. Maréchal et al. (2004) reported, based on pumping tests in a crystalline hard-rock aquifers in India, fractured zones of mean hydraulic conductivity 100 to 400 times greater than that of the surrounding rocks.

#### Model conceptualisation and setup

At first, the available hydrogeological data of the LRB were compiled and synthesised into a hydrogeological conceptual model. Data included both existing geological and hydrogeological information and expert and local people’s knowledge collected through stakeholders’ workshops with inputs from local, national, and transboundary governance actors.

On the basis of the above geological information (***Geological environment of the LRB***), and the validation and additional inputs from stakeholder discussions, a uniform total aquifer thickness of 120 m was considered for the combined three layers. The upper layer (top 12 m below ground) represented the alluvial sedimentary aquifer in valley bottoms, and the basement of the study area consisted of two aquifers: a low-yielding weathered aquifer (12–50 m below the ground) and a high yielding fractured lower aquifer below the weathered zone that extends up to 120 m below the ground.

The groundwater flow model was structured in MODFLOW-2005 using three layers according to the conceptual model considering the main hydrogeological domains of the LRB, aquifer layering and structural discontinuities, including major faults and fracture zones (CSIR 2003; W-E cross-section) (Fig. 3). All layers are considered convertible layers between confined and unconfined depending on the conditions of the upper layer. The new simplified cross-section was developed using the existing cross-section from CSIR (Council for Scientific and Industrial Research), along with borehole lithological data collected from SADC and through discussions with local stakeholders during the stakeholders meeting.

The model area (415,000 km^2^) was discretised into a total of 773,544 smaller cells having 772 rows and 1002 columns with a grid cell dimension of 1000 m × 1000 m; cells falling outside the basin were considered inactive. As a result, in total the model comprised 2,320,632 cells (3 layers × 772 × 1002 cells) (Fig. S2 of the ESM). The digital elevation model (DEM, Table S2 of the ESM) was used to define the top of the model domain.

A no-flow boundary was considered on all lateral facies and bottom of the model except for the basin outlet. A constant head boundary equal to sea level elevation (0 m) was assigned to the basin outlet where the Limpopo River meets the Indian Ocean near Xai-Xai in Mozambique. The no-flow boundary at the bottom of the model domain was based on the assumption that beyond 120 m depth, the basement rocks are relatively impermeable and groundwater flow is negligible. The static groundwater levels obtained from the Southern African Development Community (SADC) were interpolated and used as model’s initial hydraulic heads. Different MODFLOW-2005 model packages have been used to represent various conceptual processes (Table S3 of the ESM).

#### Model parameterisation

The range of values of hydraulic properties (e.g. hydraulic conductivity, specific yield, specific storage, riverbed’s hydraulic conductance, etc.) for different geological environments was selected on the basis of the available geological information, typical values for aquifer materials, and previous research findings in the study area (Table S4 of the ESM). Forty relevant academic and grey literature documents containing in situ measurements of the subsurface hydraulic properties of the LRB have been reviewed and synthesised. For the first layer of the model, the GLobal HYdrogeology MaPS 2.0 (GLHYMPS 2.0) product (Huscroft et al. 2018)—after improvement and evaluation using in situ measurements from 40 reviewed studies—was used to represent the spatial distribution of hydraulic properties. For the second and third layers, a combination of model conceptualisation, expert knowledge, and the GLHYMPS 2.0 product, improved and updated with in situ measurements, was used.

*Hydraulic conductivity:* The calibration process yielded the greatest hydraulic conductivity values for the unconsolidated/poorly consolidated sedimentary materials, and the lowest values for the basement rocks (Table 1, Fig. S1 of the ESM). These values are in line with previously published values (Table S4 of the ESM). In basement areas, the optimised horizontal hydraulic conductivity for the third (lowest) layer reflecting the fractured basement, i.e. the saprock (0.51 m/d) was greater than the values for the second (middle) layer reflecting the weathered basement, i.e. the saprolite (0.34 m/d). These results are consistent with other studies in the LRB (Jolly 1986; Holland 2011, 2012; Ebrahim et al. 2019; Abiye et al. 2020).

**Table 1:** Range of hydraulic properties optimised values for different layers and hydrogeological regions, including the sources of initial values used

Parameters	Descriptions	Unit	Basement	Volcanic/sedimentary	Consolidated	Consolidated, intergranular	Unconsolidated/poorly consolidated
Horizontal hydraulic conductivities (hk)	Ranges	m/d	0.003–2.5	0.003–5.18	0.1–11.7	1–15	10–700
	Sources	Table S4 of the ESM					
	hk_l1: hk of layer-1	m/d	0.52	1.30	2.73	8.66	133.81
	hk_l2: hk of layer-2	m/d	0.34	1.09	1.64	6.15	108.72
	hk_l3: hk of layer-3	m/d	0.51	1.36	3.11	-	5.184 (consolidated)
Specific storage (ss)	Ranges	m^−1^	2.1×10^−6^−5.7×10^−2^	2.1×10^−6^ − 5.7×10^−2^	2.1×10^−6^ − 5.7×10^−2^	2.1×10^−6^ − 5.7×10^−2^	2.1×10^−6^ - 5.7×10^−2^
	Sources	Batu 1998; Holland 2011; Staudt 2003, 2004; Rushton and Weller 1985; Ebrahim et al. 2019					
	ss_l1: ss of layer-1	m^−1^	4.5×10^−5^	3.13×10^−4^	6.5×10^−4^	8.5×10^−4^	1.5×10^−3^
	ss_l2: ss of layer-2	m^−1^	4.5×10^−5^	3.13×10^−4^	6.5×10^−4^	8.5×10^−4^	1.5×10^−3^
	ss_l3: ss of layer-3	m^−1^	2.25×10^−5^	1.57×10^−4^	3.25×10^−4^	4.25×10^−4^	7.5×10^−4^
Specific yield (sy)	Ranges	−	0.01–0.25	0.02–0.26	0.02–0.26	0.02–0.26	0.10–0.35
	Sources:	Rusinga and Taigbenu 2005; Owen and Dahlin 2005; Moyce et al. 2006; Love et al. 2007, 2011; DWA 1991; Blok 2017; Ebrahim et al. 2019; Abiye et al. 2020; Saveca 2022					
	sy_l1: sy of layer-1	-	0.05	0.14	0.20	0.15	0.27
	sy_l2: sy of layer-2	-	0.01	0.09	0.19	0.12	0.25
	sy_l3: sy of layer-3	-	0.05	0.14	0.20	0.15	0.27

Regional dykes and faults were calibrated with hydraulic conductivities of around 25 to 38 times greater than the surrounding basement and around 5 to 12 times greater than the surrounding consolidated, sedimentary, and volcanic rocks (Fig. S1 of the ESM). These values are consistent with other regional studies in basement rock aquifers (e.g. Dippenaar 2008; Maréchal et al. 2004; Abiye et al. 2020).

Vertical hydraulic conductivity was assumed to be 1/10 of the horizontal hydraulic conductivity in unconsolidated sediments and consolidated sedimentary rocks as commonly used in modelling studies. In the basement, as well as for regional dykes and faults, horizontal and vertical hydraulic conductivity were assumed to be equal.

*Specific storage and specific yield:* The greatest specific storage values were obtained for unconsolidated/poorly consolidated sedimentary materials, while the smallest values were obtained for the basement aquifers (Table 1). Although unconfined conditions likely prevail in the shallow subsurface represented by layer 1 (L1), the model considered all layers to be convertible between confined and unconfined depending on the conditions of the upper layer. This is why the same specific storage value was applied for both layer 1 and layer 2. In the basement, the calibrated specific storage for the third layer was lower than the values for the first and second layer (Table 1). Although the calibrated range of specific storage values for the basement falls within the upper range of typical values for fractured rocks (3.3 × 10^−6^ to 6.9 × 10^−5^ m^−1^) (Batu 1998), these results are consistent with other studies from the LRB (Holland 2011; Ebrahim et al. 2019). The calibrated specific yield values are also within the range of the values published for studies across the LRB. For the basement rocks specifically, specific yield values for layer 3 were a bit greater than the values in layer 2 (Table 1). Ebrahim et al. (2019) also reported similar findings from their study in South Africa.

*Rivers and drains:* The main Limpopo River is perennial, and most of the basin’s small rivers are ephemeral with very little to no flow during the dry period. The major, mostly perennial rivers across the LRB (Fig. S2b of the ESM) were simulated in the model using the River package based on the available river discharge data (Table S2 of the ESM). All smaller ephemeral distributaries and tributaries (Fig. S2b of the ESM) were represented with the Drain package of MODFLOW. The riverbed’s hydraulic conductances (${C}_{\mathrm{r}\mathrm{i}\mathrm{v}}$) were assigned as a function of the model grid size and the riverbed’s hydraulic conductivity and thickness, using the following equation:

1 $${C}_{\mathrm{r}\mathrm{i}\mathrm{v}}=\frac{{K}_{\mathrm{r}\mathrm{i}\mathrm{v}}{W}_{\mathrm{r}\mathrm{i}\mathrm{v}}{L}_{\mathrm{r}\mathrm{i}\mathrm{v}}}{{d}_{\mathrm{r}\mathrm{i}\mathrm{v}}}$$

where ${K}_{\mathrm{r}\mathrm{i}\mathrm{v}}$ is the riverbed’s vertical hydraulic conductivity [L T^−1^], ${d}_{\mathrm{r}\mathrm{i}\mathrm{v}}$ is the thickness of the riverbed (assumed to be 1.5 m), ${W}_{\mathrm{r}\mathrm{i}\mathrm{v}}$ is the width of the river within the model grid cell [L], and ${L}_{\mathrm{r}\mathrm{i}\mathrm{v}}$ is the river’s length within the model grid cell [L].

The riverbed’s vertical hydraulic conductivity was considered to be one-tenth of the horizontal hydraulic conductivity of the unconsolidated alluvial deposit (typically sandy). The range of initial riverbed conductivities was assessed on the basis of the measured and estimated values across the LRB (Table S4 of the ESM). The calibrated riverbed’s vertical and horizontal hydraulic conductivity were 13.381 and 133.81 m/d, respectively. These values fall within the ranges of published values across the LRB (Moyce et al. 2006; Walker et al. 2018; Saveca et al. 2022).

Mpala et al. (2016) and de Hamer et al. (2008) measured the thickness of alluvial sands in Zimbabwe using probing methods and reported thicknesses 1.5 and 1.4 m. Walker et al. (2018) measured the sand thickness of between 0 and 2 m in the Molototsi River in the Limpopo province of South Africa using trial pitting and probing methods. Other studies across the LRB also reported sand thickness values in sand rivers varying between 0.5 and 10 m (Walker et al. 2018, and Saveca et al. 2022 for more details). On the basis of this information, the thickness of the riverbed was considered to be 1.5 m in this study.

River stage data across the LRB are not available. River stage and other parameters are computed from river discharge data and the DEM, with the assumption that river depth is proportional to the river discharge. The river discharge records were collected from the Global Runoff Data Centre (GRDC) (Table S2 of the ESM). On the basis of this model, the depth of the rivers was calculated to vary from 1.65 to 7.66 m across the LRB.

#### Groundwater recharge simulation

The spatially distributed monthly groundwater recharge in the LRB was simulated from 2009 to 2019 using a spatially distributed water balance model, WetSpass (Batelaan and De Smedt 2007; Abdollahi et al. 2017). WetSpass is a GIS-based spatially distributed semi-physical model in which groundwater recharge is calculated as a residual of the water balance for each model cell. The long-term average monthly groundwater recharge was initially used as input of the steady-state groundwater model, while monthly groundwater recharge timeseries were used as time-varying inputs for the transient groundwater model. The WetSpass model considers land cover heterogeneity by dividing every cell into four fractions: bare soil, open water, impervious, and vegetated. Spatially distributed maps of elevation, land cover, soil texture, groundwater depth, precipitation (including rainy days), evapotranspiration, temperature and wind speed have been used as inputs for WetSpass (Table S2 of the ESM). Spatially distributed matrixes of 1000 m × 1000 m resolution were prepared using ArcGIS and Python for all inputs. WetSpass was further calibrated and evaluated by coupling it with MODFLOW-2005 using FloPy package through minimising the misfit between observed and simulated groundwater levels.

#### Groundwater abstraction estimation

Groundwater abstraction was estimated from two sources: (i) recorded borehole yield of 10,755 boreholes across the LRB collected from SADC Groundwater Information Portal (Fig. S2d and Table S2 of the ESM), and (ii) recorded licensed abstraction rates of wells within South Africa collected from SADC Groundwater Information Portal (Fig. S2d and Table S2 of the ESM). It is estimated that the datasets represent approximately 80% of existing boreholes for both yields and licensed rate, and these were considered as a good representation of groundwater abstraction across the LRB, according to consulted stakeholders’ (e.g. SADC-GMI, International Water Management Institute, LIMCOM, Department of Water and Sanitation in South Africa, Department of Water Affairs in Botswana, Ministry of Energy, Water & Climate in Zimbabwe, Dabane Trust in Zimbabwe, Irrigation Department Zimbabwe, and ARA-Sul in Mozambique), as there is no systematic record of actual abstraction across LRB. For the steady-state model, the collected and available steady rates were used, assuming their equivalence to the rates in 2009. For transient multi-annual modelling, an increase in groundwater abstraction was calculated, assuming a direct correlation between growing population and groundwater abstraction. Owing to limited data availability, the increase in abstraction rate is only considered in the existing wells, without considering the spatial expansion of the number of wells, which could also be the case in the future. Data sources for population density and demographic increase data are presented in Table S2 of the ESM.

#### Model calibration and performance evaluation

Groundwater level observations are used to assess the performance of the model. Long-term static water levels of 12,241 boreholes (Fig. S2c of the ESM) across the LRB were used to calibrate the steady-state model. In contrast, 120 observation wells (Fig. S2c of the ESM), all within South Africa had temporal records of water level which were used to calibrate the transient model. No time series of observed groundwater levels was available for the other three countries. Model parameters were estimated using manual trial-and-error calibration through (i) using a graphical presentation of the observed and simulated level and (ii) aiming at producing optimal values of statistical indicators (Table 2) based on minimal misfit between observed and simulated levels. Calibrated parameters are listed as follows: riverbed hydraulic conductance, horizontal hydraulic conductivity, vertical hydraulic conductivity, specific storage, and specific yield of different geological environments. The ranges, initial values, and optimised values of the parameters are summarised in Table 1.

**Table 2:** Statistical indices used for model performance evaluation

Model	Number of observations	*R*^2^	RMSE (m)	rRMSE (m)	ME (m)	MAE (m)	PBIAS (%)	NSE	KGE
Steady-state model	12241	0.987	37.523	3.850	−24.154	28.487	−2.636	0.978	0.930
Transient model	16673	0.978	46.01	5.302	−0.503	31.193	−0.058	0.977	0.946

*R*^2^ coefficient of determination, *RMSE* Root mean squared error; *rRMES* Relative root mean squared error; *ME* Mean error; *MAE* Mean absolute error; *PBIAS* Percent bias; *NSE* Nash-Sutcliffe efficiency; *KGE* Kling–Gupta efficiency

### Workshops: Identification and evaluation of management scenarios and co-creation of management recommendations

Several regional and transboundary workshops and several one-to-one discussions were conducted with different stakeholders (Table S1 of the ESM), to identify feasible management scenarios that aim: (i) to reduce impacts and increase resilience to flood–drought cycles; and (ii) to increase groundwater sustainability throughout the basins.

Through this co-creation approach, the following water management scenarios were identified: (1) managed aquifer recharge (MAR), using (1a) injection well(s) next to large dams capturing excess water, (1b) rainwater and runoff harvesting (RRH) and infiltration through local wells, (1c) RRH through local ponds, and (1d) small water reservoirs (e.g. sand dams); (2) deforestation; (3) afforestation; and (4) increase of groundwater abstraction: (4a) 1.25 times, (4b) 1.5 times, and (4c) 2 times. The management scenarios were run over the period 2009–2019 as a current situation and the period 2025–2050 as a future projection. After running the agreed scenarios, the management scenarios were evaluated through discussion of model outputs during another series of several regional workshops, and one-to-one discussions involving a representative sample of the stakeholder met before (Table S1 of the ESM). This allowed for (i) refining the identified scenarios, and (ii) further improving the model conceptualisation where required, and finally (iii) identifying feasible regional management scenarios.

The identified scenarios through this iterative co-creation approach have been evaluated using the calibrated model, and the results of different scenarios are summarised and presented in the results section.

#### Increase groundwater abstraction

Population data shows that population is growing in the four countries of the LRB, and as a result it was estimated that population density by 2050 may increase to up to 2.6 times the 2010 population density in different parts of the basin. Water supply is also increasing accordingly to meet the additional demand. Literature shows that irrigated surfaces are also increasing in the LRB. Jolly (1986) reported that groundwater abstraction for irrigation in parts of the LRB in South Africa doubled in just 18 years. However, groundwater abstraction may not increase proportionally with the population due to improving agricultural water use efficiency and the introduction of an effective groundwater abstraction policy in the future, which is currently missing. Considering all those factors and stakeholders’ suggestions, three different groundwater abstraction scenarios were considered. These scenarios assume that groundwater abstraction will increase at three different rates, with abstraction reaching, by 2050 values of (i) 1.25 times, (ii) 1.5 times, and (iii) two times the current groundwater abstraction. The impact of increased abstraction was then evaluated by spatially comparing the simulated groundwater levels using (a) current abstractions and (b) increased abstractions through subtracting future simulated water levels minus present simulated levels across the basin, hence displaying spatial distribution of change in groundwater level, i.e. increase/decrease when positive/negative sign, respectively.

#### Managed aquifer recharge (MAR)

The impact of MAR was evaluated in four different ways: using (i) injection wells next to large dams, (ii) rainwater harvesting (local wells), (iii) rainwater harvesting (local ponds), and (iv) small water reservoirs (e.g. local ponds and sand dams). These scenarios help evaluate the feasibility of capturing additional water causing floods during the rainy season and storing it in the subsurface to mitigate the impacts of subsequent droughts on water availability for human and wider ecosystems.

*Injection wells adjacent to large dams*: The long-term average of daily reservoir outflow was considered as the normal outflow. The injection wells were simulated close to the outlets of major dams across the LRB using dam outflow above the normal as MAR, assuming that outflow above the normal flooding around the dam area. This approach focused primarily on water sources and stakeholder preferences to mitigate the negative impacts of floods. Figure S3 of the ESM shows the dam locations across LRB as implemented in the model. The most recent data (Table S2 of the ESM) available for the past 12 years were used as input, given the varying availability of outflow data across different dams.

*Rainwater and runoff harvesting (RRH) through small-scale water reservoirs/local ponds, local wells and sand dams*: In these scenarios, the part of the rainfall and runoff that contributes to flooding was captured through small-scale water reservoirs/local ponds, local wells and sand dams for subsequent infiltration and subsurface storage for use during droughts.

To evaluate the impact of RRH through small-scale (household to village) water reservoirs/local ponds and local wells, the weighted fraction of spatially distributed runoff simulated using WetSpass was used as additional recharge to existing groundwater recharge. For this, the runoff exceeding normal levels was used, assuming that runoff exceeding normal levels contributes to flooding, while normal runoff is considered to represent natural flow and was not captured to maintain streamflow. The long-term monthly average runoff for each cell was taken as the normal runoff. However, it was assumed that a maximum of 40% of the above-normal runoff can be stored underground for small-scale water reservoirs, local ponds and sand dams, assuming 60% evaporation losses. It has been observed that average evaporation from open water varies from 50 to 78% of the rainfall for different months. Jovanovic et al. (2015), Chaudhuri (2021) and IWMI (2022) also reported that evaporation from open water bodies and reservoirs in arid and semi-arid climates can be significantly great and may range from 30% to 90% of the rainfall. The population density data (Fig. 1d) were used to assign the weight (Table S5 of the ESM), assuming that aquifer storage capacity is not limiting, i.e. that more water can be stored underground where population density is greater. Population density influences RRH implementation in two ways: higher density provides more existing infrastructure for small-scale use and a larger community base to support new developments. This assumption was based on the preliminary data analysis, which indicated that during dry seasons, aquifers have enough storage capacity available to store extra water.

Local wells were similar to ponds, but they were assumed to be not subject to evaporation since the water was infiltrated directly in the subsurface through the well. However, unlike injection wells, local wells did not involve high pressure injection.

Following the local ponds, the effect of small water reservoirs and sand dams built along the tributaries was also evaluated using the weighted fraction of spatially distributed above-normal runoff excluding evaporation (40%) as additional recharge to existing groundwater recharge. Again, the population density data (Fig. 1d) were used to assign the weight (Table S5 of the ESM) assuming greater dam density in greater population areas. However, this adjusted runoff has been applied only across the tributaries (Fig. S2 of the ESM) setting on sandy or loamy soil types, as sand dams were assumed most effective in well-drained soils favouring subsurface infiltration.

#### Land use–land cover changes

The impact of land use–land cover changes was evaluated using the following two scenarios: (i) the impact of deforestation, and (ii) the impact of afforestation.

Ngcobo et al. (2013) suggested that land use change has a greater impact on water systems than climate change in southern Africa. Rapid population growth and economic expansion have resulted in extensive land use and land cover changes. For example, the growth of cities has led to increased demand for fuelwood and charcoal, resulting in deforestation in surrounding areas (Ngcobo et al. 2013). On the other hand, Mkwalo (2011) reported that there is substantial potential for afforestation in the basin, similar to stakeholders’ suggestions. For example, the Limpopo province has suitable conditions for forestry plantation, but its current plantation area is only 4.7% of South Africa’s total (Mkwalo 2011).

To evaluate the impact of deforestation, after discussing with stakeholders, a scenario was considered in which 50% of the mixed forest areas of the basin (which constitute 11.6% of the total area: Fig. 1c) would convert to bare areas in the future due to deforestation. On the other hand, to assess the impact of afforestation, a scenario was considered in which bare areas and 50% of shrub-covered areas would convert to mixed forest areas in the future as a result of afforestation.

## Results

### Model performance and challenges under data-limiting conditions

**Fig. 4:**
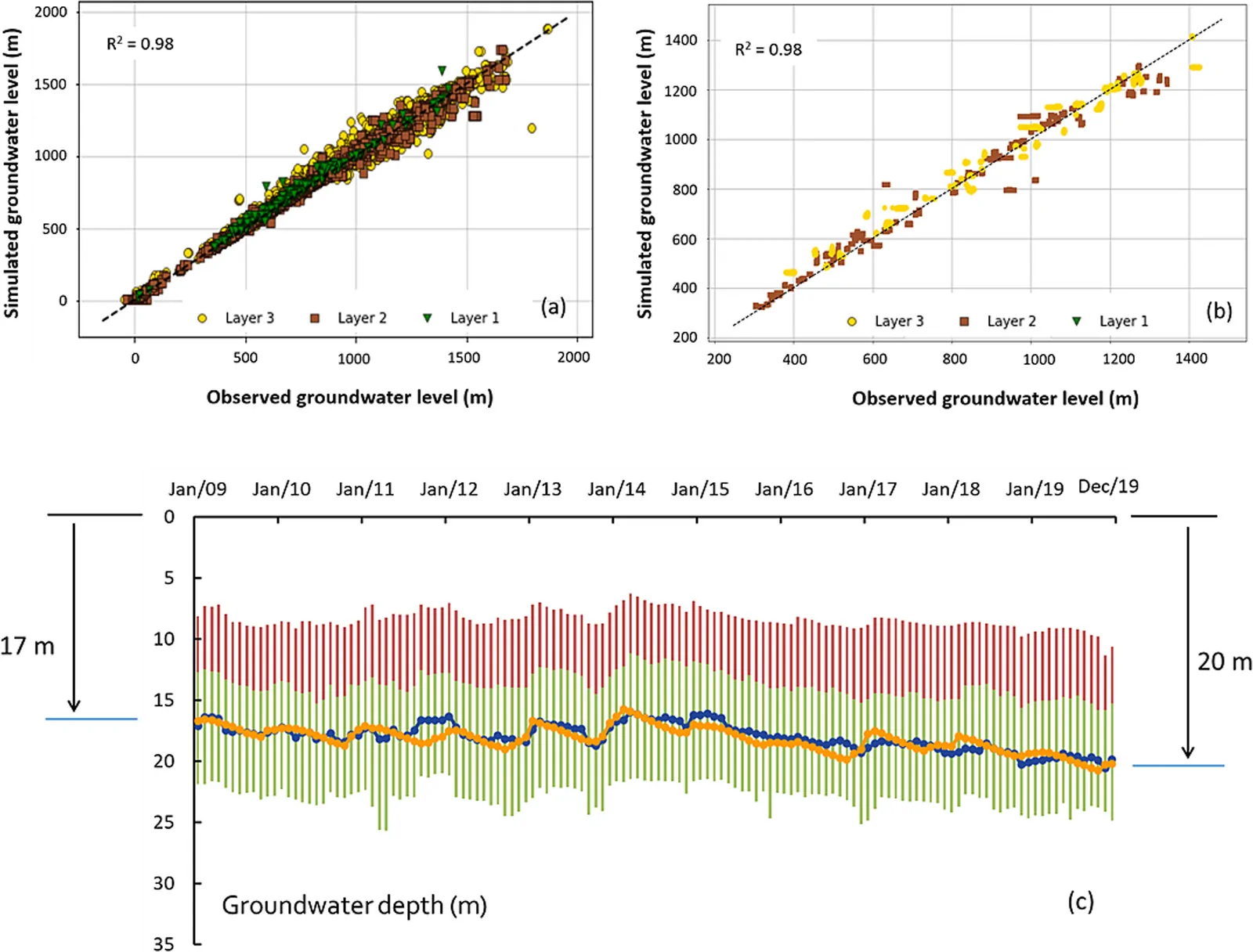
Scatterplot of simulated versus observed levels for the steady-state model (a) and transient model (b). (c) Groundwater depth box plot from 2009 to 2019, which combines the monthly ranges and average of all available groundwater level observations across the basin. The blue line indicates the observed average depth, while the observed 25th and 75th percentiles are represented by the red and green bars, respectively. The yellow line indicates the simulated average depth

The scatterplot for both the steady-state and transient models show that the observed and simulated levels are close to the 1:1 line, indicating that model simulation outputs agree reasonably well with the observation datasets for the spatial scale considered (Fig. 4 a, b).

The transient model shows a clear performance improvement compared to the steady-state model; however, the number of observations used to evaluate the transient model (120) is significantly less than the steady-state model (12,241).

Statistical indicators confirm the acceptable simulation capacity of the model (Table 2). The values of Nash–Sutcliffe efficiency (NSE 0.98), Kling–Gupta efficiency (KGE 0.95) and relative root mean squared error (rRMSE 3.85) show the excellent simulation capacity of the model in simulating groundwater levels at the locations of the observation points. The negative values of Percent bias (PBIAS) indicate that model is slightly overestimating groundwater levels. However, residuals are distributed around the zero line but not equally spread.

Although residuals decrease for the transient model, they remain significant for a few observation wells. In most cases, the same well is used for abstraction and observation, increasing data uncertainty. Additionally, the limited availability of groundwater abstraction data might be the reason for the high residuals at several observation wells. Owing to lack of data on the actual well/borehole abstraction and their spatial and temporal distribution, reported borehole yields were applied as groundwater abstraction, which likely overestimates the actual groundwater abstraction. A simplified representation of the river system may be another reason for the significantly great residuals.

However, the simulated groundwater level depths were in good agreement with the observed groundwater level depth between 2009 and 2019 (Fig. 4c). Both the observed and simulated data show an increasing trend in groundwater level depth, i.e. a decrease in water level elevation. Both yearly and seasonal variations of the groundwater level depth were also well captured by the simulated series. This further demonstrates the model’s acceptable simulation capacity.

### Spatio-temporal hydrological connectivity

**Fig. 5:**
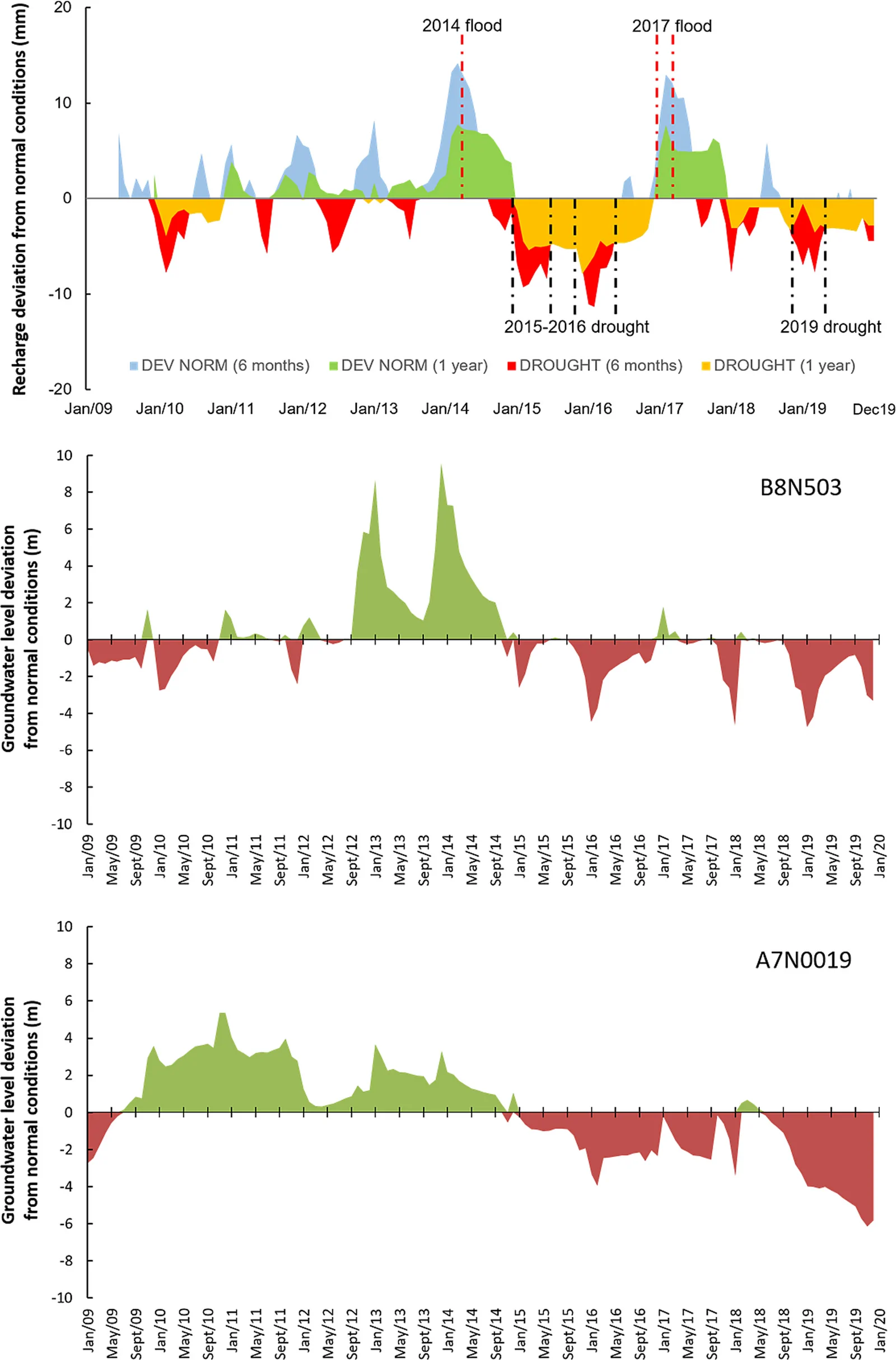
Simulated groundwater recharge deviation from the normal (a) and groundwater level (two selected observation wells, B8N0503 (b) and A7N0019 (c), are shown in Fig. S2c of the ESM. Here normal means long-term average. DEV NORM (6 months and 1 year) in the top picture represents deviations above normal (positive anomalies) of the 6-month and yearly averages, respectively, and DROUGHT (6 months and 1 year) represents deviations below normal (negative anomalies) of the 6-month and yearly averages, respectively. The dotted lines of floods and droughts represent the historical records of floods and droughts

Figure 5 shows that the simulated groundwater recharge deviations from WetSpass modelling match the historical records of hydrological extreme events (floods and droughts). Overall, across the basin, groundwater recharge is greater than normal during flood events and less than normal during droughts. Normal is defined as the long-term monthly average. Negative and positive values indicate drought and flood events, respectively. For example, 2015–2016 recorded a 2-year drought of the driest conditions in 35 years over the entire LRB (Lindevall 2017; LIMCOM 2021). This is reflected by recharge model outputs, where groundwater recharge deviation also shows negative values for the period 2015–2016 (Fig. 5).

Simulated groundwater recharge anomalies were positive in January, February and March 2017 during which Franchi et al. (2024) also reported flood events during that period. This agrees with the deadly floods reported during that period, which killed 246 people in southern Zimbabwe and cost around 100 million USD (BBC 2017). Significant country-scale floodings were also reported in South Africa and Botswana (Reliefweb 2017).

Enhanced recharge associated with reported major flood events resulted in increased simulated groundwater levels as reflected by the observed positive groundwater level deviation to long-term average levels. In contrast (Fig. 5), reduced groundwater recharge associated with prolonged droughts resulted in decreased groundwater levels, as shown by negative deviations to long-term groundwater level average. Thus, groundwater level anomalies closely relate to groundwater recharge anomalies associated with major historical floods and droughts. However, the impact is not uniform across all locations. For instance, there seems to be a correspondence between groundwater recharge anomalies and groundwater level anomalies in B8N503, but not as much for A7N0019. It appears that B8N503 groundwater levels show greater correlation with shorter timescales of events (floods and droughts), while A7N0019 groundwater levels are more correlated with longer timescales. This might be because A7N0019 is located upstream in an already stressed sub-catchment where groundwater depth is relatively deeper. On the other hand, B8N503 is located downstream where groundwater depth is relatively shallower, and groundwater–surface water interaction is also relatively greater.

Although short-term groundwater level fluctuations were clearly influenced by floods and droughts, longer-term plots of groundwater level depth showed an overall declining trend suggesting that groundwater storage has been slowly decreasing in the LRB over the past decade (Fig. 4c). The spatially averaged groundwater depth below ground level was 17 m in 2009, which increased to 20 m depth in 2019 (Fig. 4c). This could be because of (1) the negative effect of recurrent droughts on groundwater recharge is more pronounced that the positive effect of recurrent floods, for example if magnitude of droughts is greater than that of floods; (2) increasing groundwater abstraction to meet the growing water demand for agriculture, household and industry, as a result of population growth and recurrent meteorological droughts; or (3) a combination of both. These findings are consistent with the stakeholders’ observations collated in this study, as well as with those reported by Jolly (1986), FAO (2004) and Ebrahim et al. (2019). These authors also reported that in some parts of the LRB in South Africa, groundwater abstraction for irrigation, livestock and domestic supply has been increasing rapidly up to exceeding the average groundwater recharge rate.

**Fig. 6:**
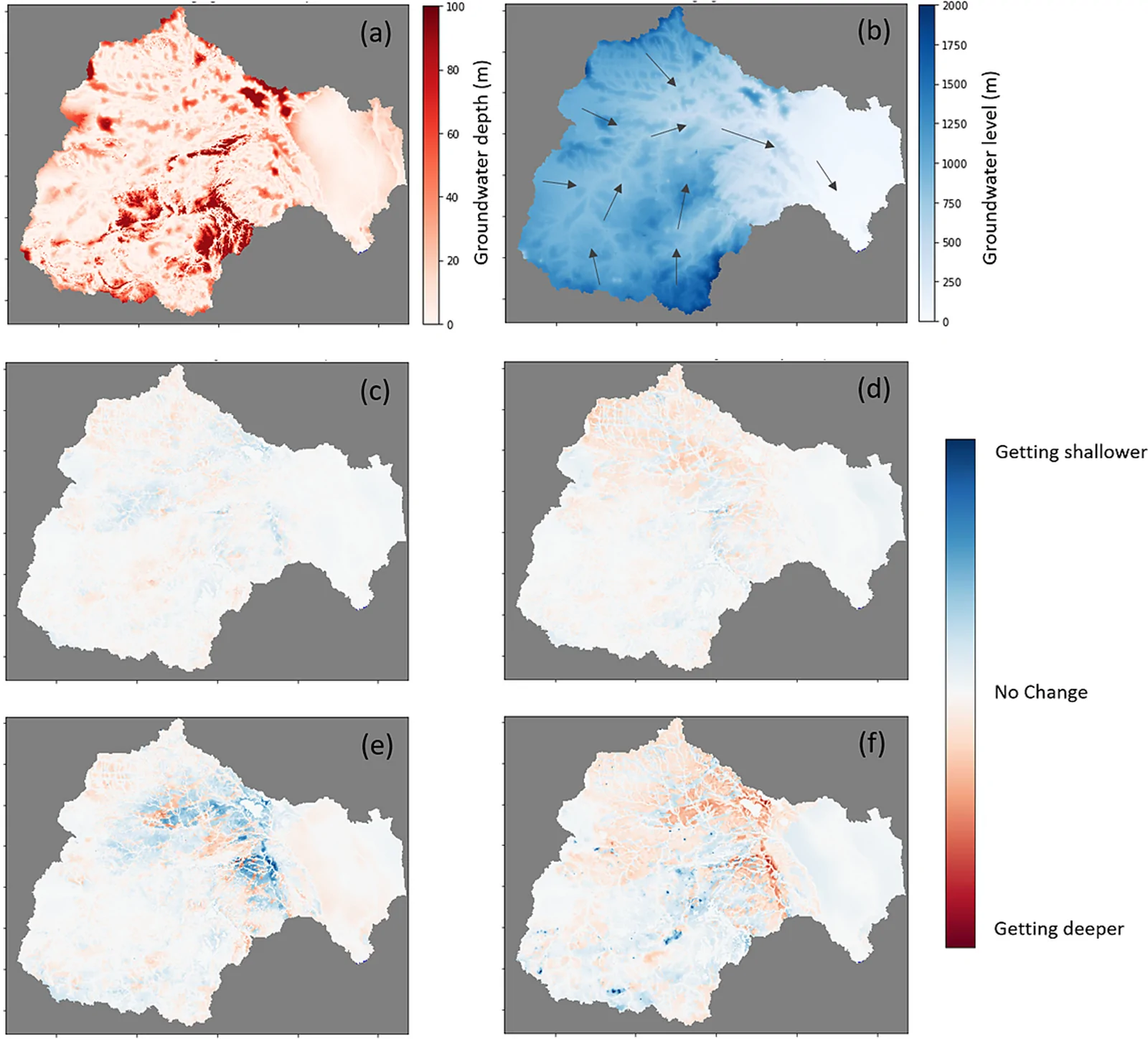
Simulated average groundwater depth (a) and groundwater level elevation (b) for period 2009–2020 in the LRB, with regional groundwater flow direction (black arrow) shown on the groundwater level elevation map. Changes in groundwater depth between normal (average) groundwater depth and short-duration floods (c), droughts (d), long-duration floods (e) and droughts (f). Short and long-duration floods and droughts are selected on the basis of Figs. 5a and 8 and indicated in Fig. 8

Simulated groundwater levels generally follow the topographic elevation, being higher upstream and lower downstream, indicative of regional groundwater flow (Fig. 6b). Simulated groundwater depths are relatively deeper in the upstream part of the basin, i.e. the upland areas where elevation is higher (Fig. 6a). Geris et al. (2022) reported for a low-productivity aquifer in the upstream part of the LRB groundwater depth that can exceed 100 m in some locations. The model findings are also in line with their observations (Fig. 6a). Both floods and droughts significantly affect groundwater depth. Extended periods of flooding notably reduce groundwater depth, whereas prolonged droughts increase groundwater depth, particularly in the upstream and central region of the basin. Similarly, short-term floods and droughts have a comparable but relatively milder impact (Fig. 6 c–f).

**Fig. 7:**
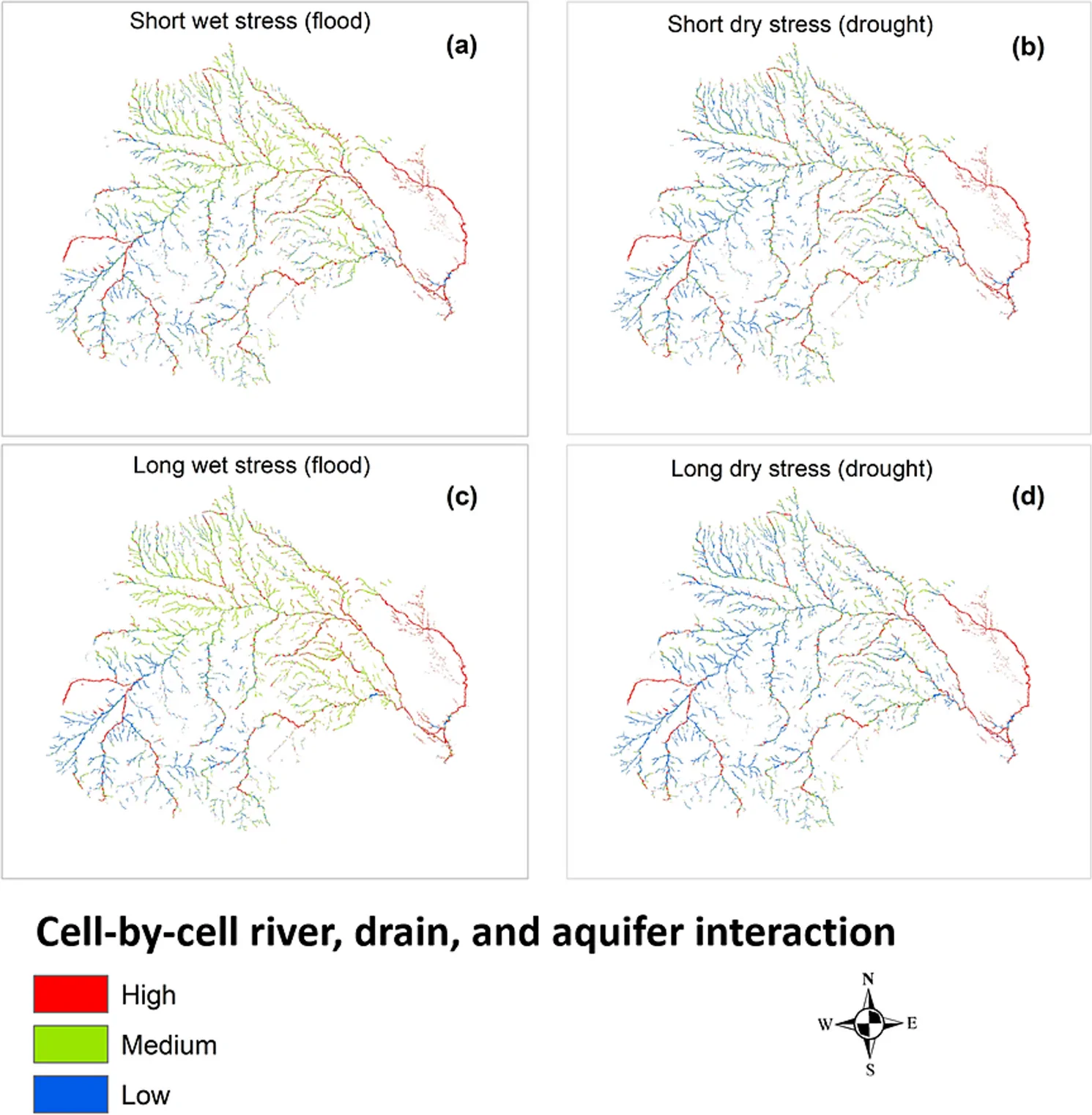
Cell-by-cell groundwater–surface water interaction/exchange in the LRB during short-duration floods (a), droughts (b), long-duration floods (c) and droughts (d). Short- and long-duration floods and droughts are selected on the basis of Figs. 5a and 8 and indicated in Fig. 8

Observations of spatially distributed simulated river and drainage leakage and/or discharge show that it varies significantly spatially across the basin. Spatial variations depend on the topography, geology, rainfall distribution and depth of the groundwater table. However, it is observed that the seasonal and multiannual fluctuation of groundwater–river exchange is significantly greater in the downstream part of the basin compared to the upstream part, where groundwater tables are shallower and rainfall is greater overall compared to upstream areas (Figs. 6 and 7). In the upstream areas where the groundwater table is deeper, streams and rivers are mostly losing water to the aquifer. These interpretations are consistent with the findings of Mosase et al. (2019).

During floods, groundwater–surface water interaction intensifies, especially in the central region of the basin (Fig. 7c–d). As a result, there is an increase in groundwater levels following floods (Figs. 6 and 7). There is no considerable difference in the groundwater–surface water interaction between short and long-duration floods and droughts (Fig. 7 a–c or b–d). However, there is a considerable impact on the change in groundwater depth between short- and long-duration floods and droughts (Fig. 6 c–e or d–f). This is because shallow groundwater responds equally to short- and long-duration floods or droughts. However, deep groundwater is more impacted by long-duration floods or droughts. It is also observed that groundwater–surface water interaction is more pronounced in unconsolidated materials compared to basement rocks.

**Fig. 8:**
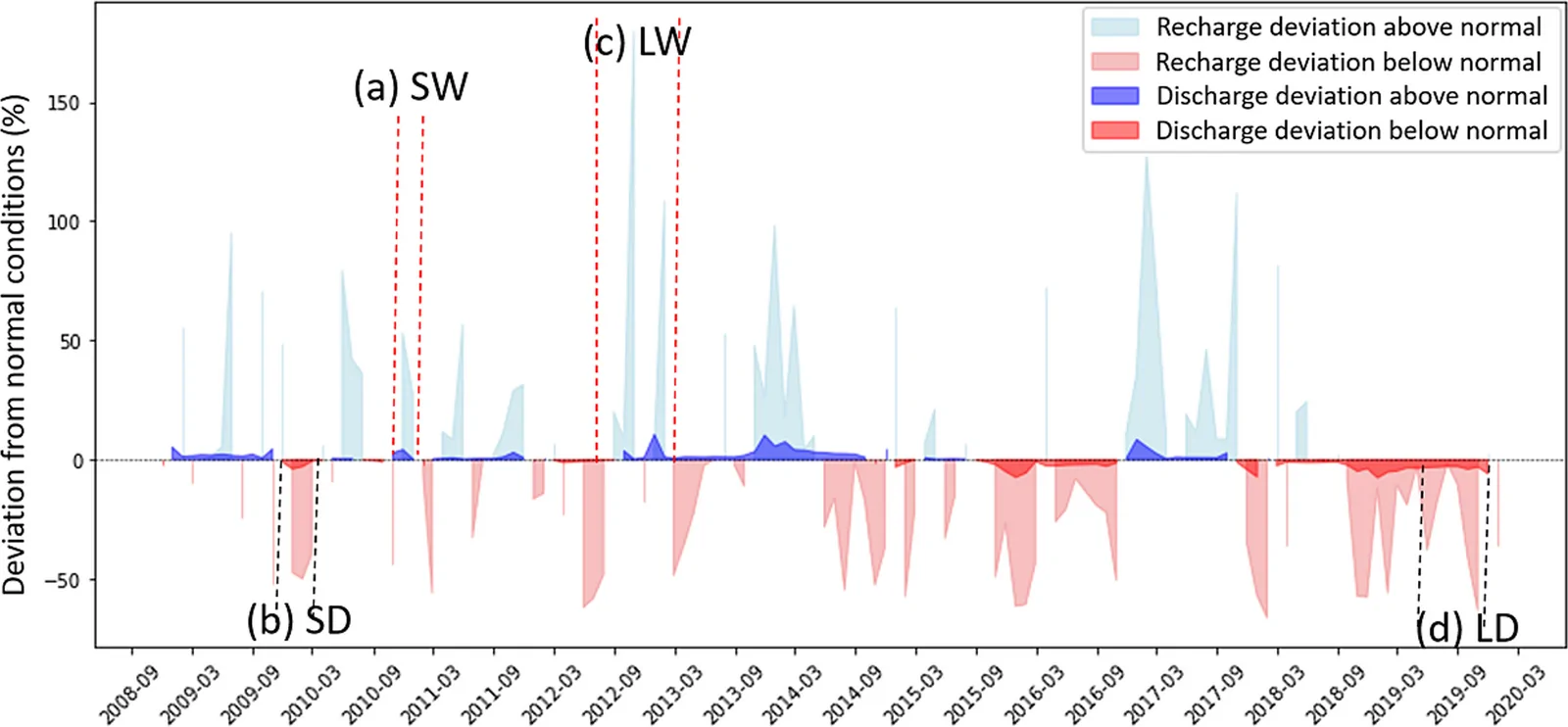
Comparison of simulated groundwater recharge and discharge (baseflow) deviation from normal. Negative deviation represents drought events, while positive deviation represents wet conditions (flood events). (a) SW, (b) SD, (c) LW and (d) LD correspond to the short-duration wet (flood), short-duration dry (drought), long-duration wet (flood) and long-duration dry (drought) conditions illustrated in Figs. 6 and 7

Groundwater recharge and discharge (baseflow) deviation from the normal show that while there is no significant shift in floods (positive anomalies) or droughts (negative anomalies) events between recharge and discharge, both the frequency (number of events) and severity (magnitude) of floods and droughts on discharge are less than those compared to recharge (Fig. 8). This reduction in frequency and severity can be attributed to attenuation in discharge through the groundwater system, highlighting the groundwater system’s crucial role in enhancing resilience to hydrological extremes. The average duration of floods and droughts on discharge shows a slight increase compared to those on recharge. Recharge from severe, long-duration floods contributes to greater and longer-lasting baseflow, as observed during 2013 and 2014. In contrast, baseflow tends to recover more rapidly after a drought when recharge occurs following periods of great rainfall. It is also observed that while individual short-duration flood or drought events have a very minimal impact on the groundwater, consecutive smaller events or long-duration events have a considerable impact on the recovery or decline of baseflow.

### Impact evaluation of management scenarios

#### Impact of increased abstraction

**Fig. 9:**
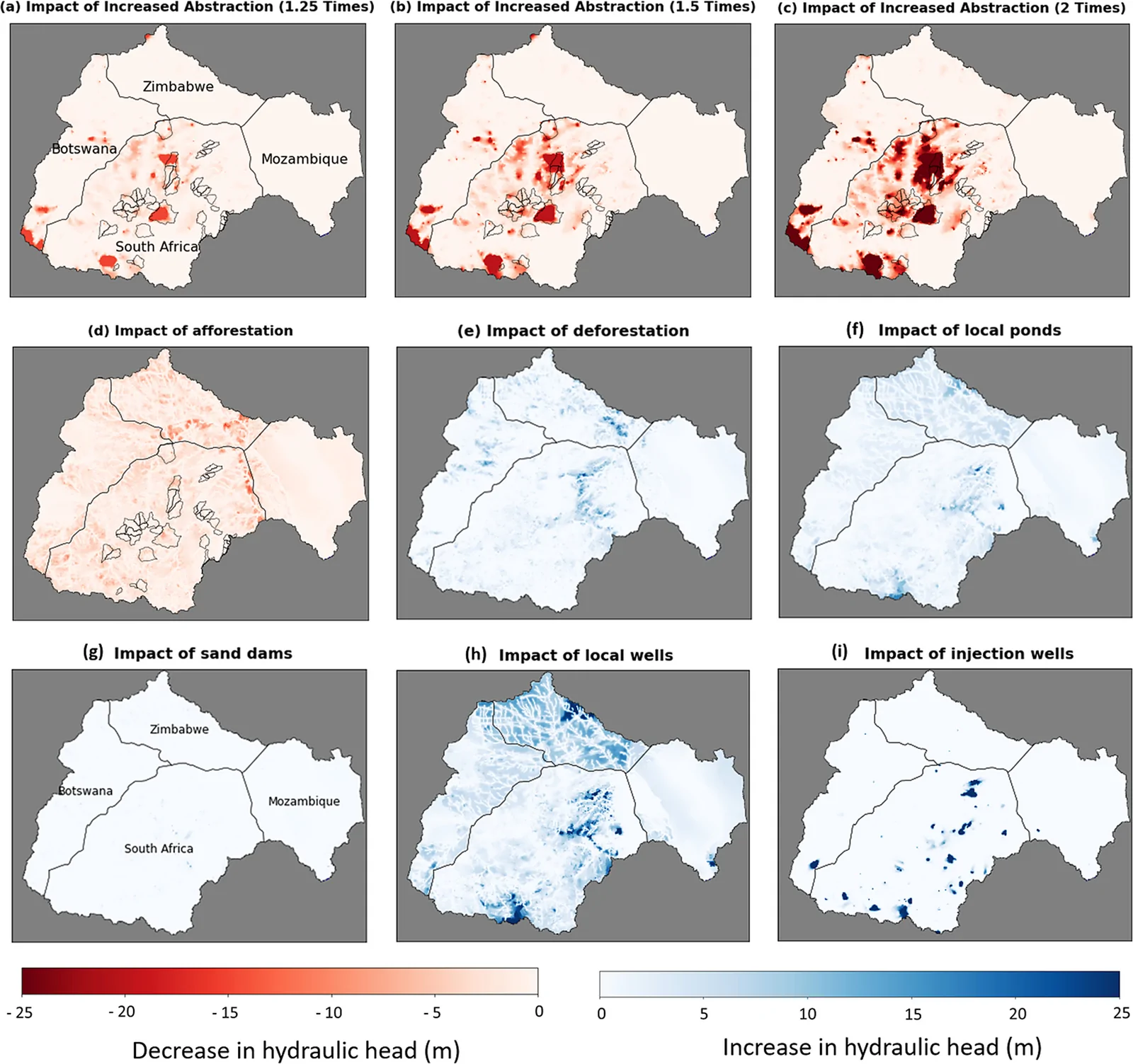
Modelled impacts of scenarios of increased groundwater abstraction, land use changes, and Managed Aquifer Recharge (MAR) in the LRB: (a)–(c) decrease in groundwater levels due to increased abstraction of (a) 1.25 times, (b) 1.5 times, (c) 2 times compared to the current, with a comparison to the location of highly water-stressed sub-catchments in South Africa (grey polygon). (d) Decrease in groundwater level due to afforestation. (e) Increase in groundwater level due to deforestation. Modelled changes in groundwater levels resulting from MAR scenarios using: (f) rainwater harvesting (local ponds), (g) small water reservoirs (e.g. sand dams), (h) rainwater harvesting (shallow local wells) and (i) injection wells next to large dams

**Fig. 10:**
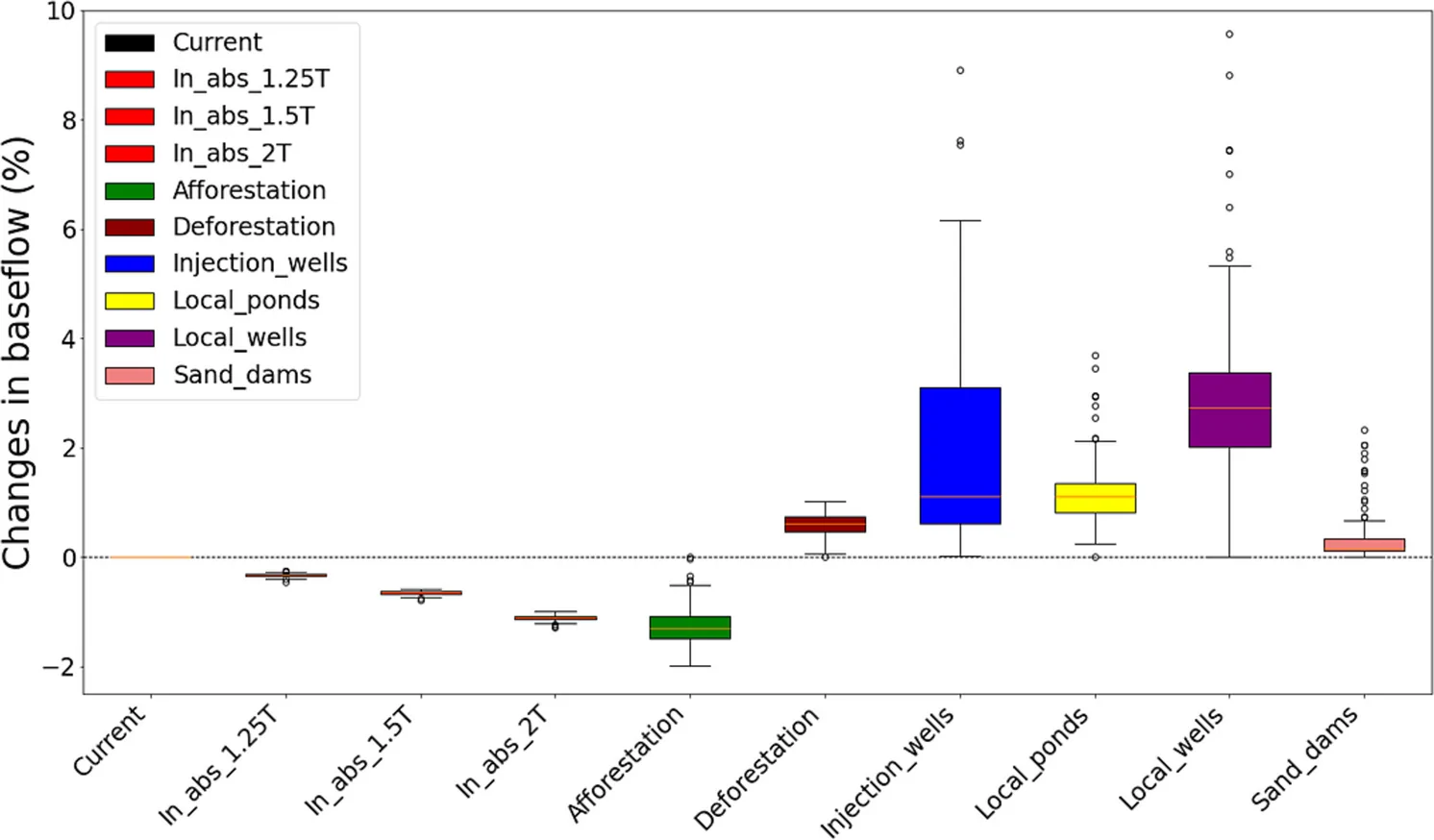
Changes in river baseflow for the different modelled scenarios, calculated cumulatively over the entire river network and over time. In_abs_1.25T, In_abs_1.5T and In_abs_2T represent increased abstractions of 1.25, 1.5 and 2 times the current level, respectively

Simulation results show that groundwater levels will continue to drop with increasing abstraction (Fig. 9). The mean groundwater level of the LRB is projected to decrease by 2, 4, and 8 m, for 1.25-, 1.5-, and 2-fold increases in groundwater abstraction, respectively. The impact is not uniform across the basin. In a few already stressed sub-catchments (highlighted in Fig. 9), groundwater levels may decrease up to 15 m even if groundwater abstraction only increases by 1.25-fold. However, if groundwater abstraction doubles, most of the stressed sub-catchments may experience a decrease in groundwater levels by up to 25 m (Fig. 9). The modelled impact of groundwater abstraction is greater in South Africa; this is because the groundwater abstraction for irrigated agriculture currently is already greater compared to the other three basin countries. It is also observed that the impacts of increased abstraction on groundwater level is higher in the already stressed sub-catchments in South Africa and the neighbouring countries (Fig. 9) because of the greater rate of increase in abstraction due to greater population growth rates. Known stressed sub-catchments are only reported for South Africa (SADC Gip 2026). It is likely that other countries have stressed sub-catchments, especially Botswana as suggested by model results showing important simulated groundwater level declines in parts of the Botswana part of the LRB (Fig. 9). The effects of increased abstraction are even greater during a prolonged drought, as groundwater recharge is less than normal during droughts (Fig. 5). Increased abstraction also increases the likelihood of groundwater drought occurrences, as it raises the magnitude of negative deviations from the normal. Increased groundwater abstractions lead to a localised decrease in groundwater head, likely due to the low hydraulic conductivity, particularly of the dominant basement geology in the basin. Increased abstraction will also decrease the baseflow (by up to 1.5%) (Fig. 10).

#### Impact of managed aquifer recharge (MAR)

*Injection wells that capture excess water from large dams:* Simulation results show that water storage through injection wells using excess water from dams during high reservoir levels and flooding events will increase the mean groundwater level in the LRB by 1.29 m. The impact is considerably greater locally for this scenario, leading to a significant increase in groundwater storage in the vicinity of the well location, where groundwater levels could locally rise by up to 25 m in places (Fig. 9i). The effect is greater around the areas where the number of implemented wells is greater. The impact also depends on the geology, available storage in the aquifer, and the distance of the injection wells from the major rivers. The impact was greater in the upstream and central parts of the basin, characterised by relatively low productivity of basement aquifers with a deeper groundwater table. On the other hand, the impact was less in the downstream part of the basin, characterised by significantly productive sediment and a shallow groundwater table. This is because aquifers with relatively lesser flow and storage capacity and a deeper water table provide less pore space but a thick unsaturated zone, accommodating the rising groundwater level. The impact was also closely spatially related to the distance of the injection well from the major rivers. This is because, if the wells are very close to the river, water flows back to the river (increased baseflow) instead of contributing to an increase in groundwater storage. For the above reasons, there was very minimal or almost no impact in the Xai-Xai area (Oxbow, Fig. S3 of the ESM) because of the proximity of the well to the river and it is characterised by greatly productive sediment and a shallow groundwater table.

**Fig. 11:**
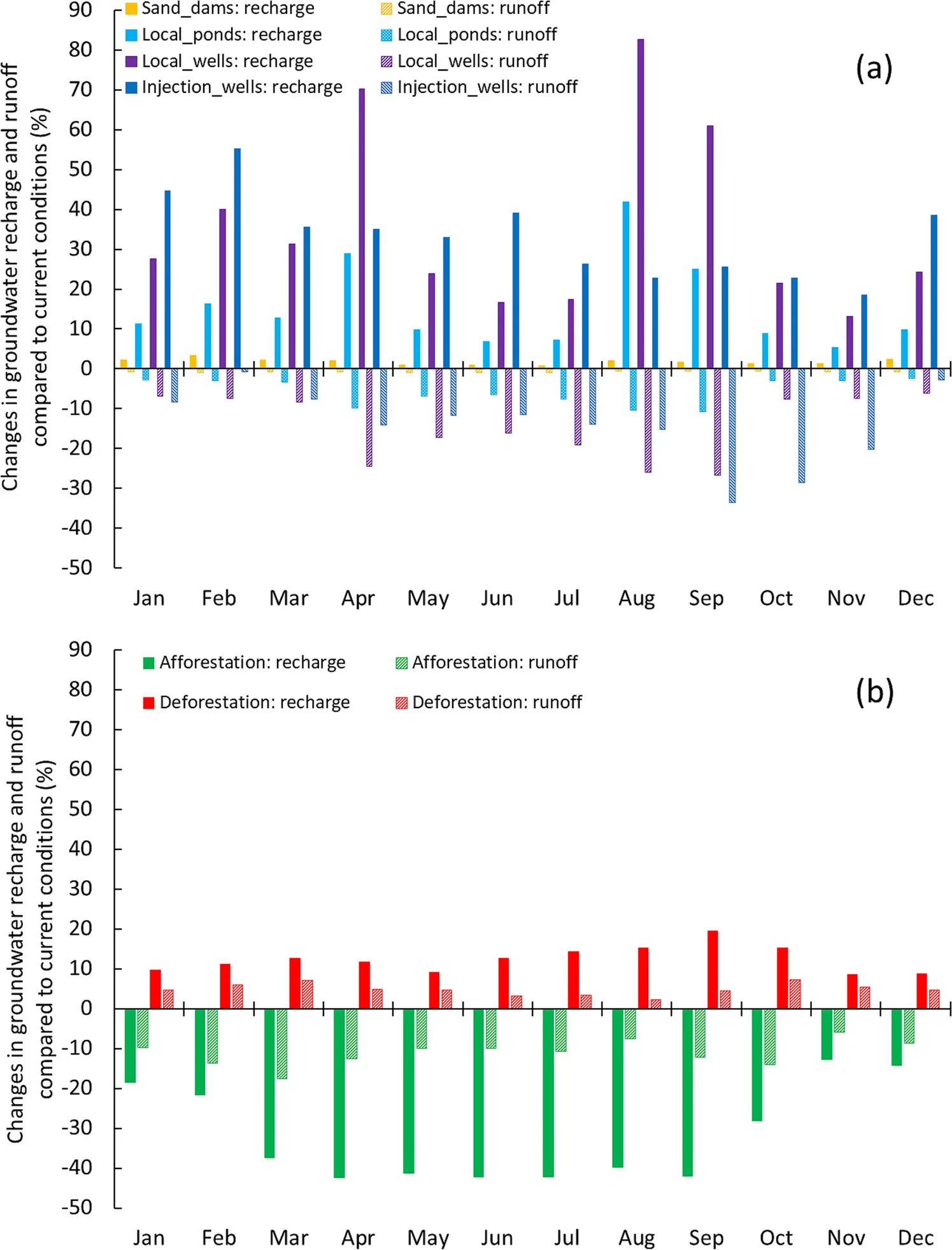
Modelled changes in groundwater recharge and surface runoff as a result of scenarios of (a) (i–iv) managed aquifer recharge (MAR) using (i) injection well next to large dams (blue), (ii) rainwater harvesting through local wells (purple), (iii) rainwater harvesting through local ponds (light blue) and (iv) small water reservoirs (e.g. sand dams) (yellow), and (b) land use–land cover changes: (v) afforestation (green) and (vi) deforestation

Injection wells could increase groundwater recharge by up to 55% (Fig. 11a), with an average increase of 33%. Injection wells could increase the baseflow by up to 6%, with an average increase of 2.5%, indicating increased baseflow to the nearby river and drainage network (Fig. 10). The baseflow is relatively greater when an injection well is proximal to the river in a significantly conductive sedimentary aquifer.

The basin-scale impact would be greater if injection wells were to be applied to all existing dams across the LRB. However, in this study, only a limited number of wells (*n*=109, Fig. S3 of the ESM) next to dams for which outflow data timeseries were available were considered, most of which are located in the South African part of the LRB.

*Impact of rainwater and runoff harvesting (RRH) through small-scale water reservoirs/local ponds, local wells, and sand dams across the Limpopo River tributaries:* Simulation results show that water storage through RRH can significantly increase groundwater levels across the LRB (Fig. 9). Among these three RRH techniques, harvesting through local wells has the greatest regional impact in the groundwater level, followed by local ponds and sand dams. This is because, unlike local ponds, local wells are not subject to evaporation, allowing more water to be stored underground. Importantly, local wells and ponds were implemented across the entire basin, based on the population distribution and density. In contrast, sand dams were applied only to ephemeral tributaries of the main Limpopo River in areas with sandy or loamy soil types, as sand dams can be implemented only in such conditions.

The groundwater recharge increased by up to 82, 41 and 3%, respectively, due to local RRH and storage through small-scale water wells, ponds and sand dams recharge (Fig. 11a).

According to the scenario simulations, the mean groundwater level in the LRB could increase by 3.19, 1.30 and 0.05 m, respectively, for RRH through local wells, local ponds and sand dams. The impact is significantly greater in areas with greater rainfall and population density, where groundwater levels could locally increase by up to 25 m as a result of the implementation of RRH and infiltration through local wells. On the other hand, groundwater levels rose only by around 10–15 m when applying infiltration through local ponds (Fig. 9). The impact of both techniques was greater in the upstream part of the basin characterised by relatively low-productivity basement aquifers (Fig. 9). This is because the aquifer has relatively less flow and storage capacity and a deeper water table, providing less pore space but a thick unsaturated zone, accommodating the increasing groundwater level. The impact was also closely related to the spatial distribution of the rainfall/recharge and population density, as a greater artificial recharge was assumed for greater population density.

Although the basin-scale impacts of flood water capture and infiltration through sand dams across the tributaries were less than RRH through local wells and ponds (Fig. 9), the simulation results suggest that sand dams can also significantly increase groundwater levels at more local scales, i.e. in the vicinity of the drainage network.

These scenarios also have positive impacts on baseflow (Fig. 10), groundwater recharge and runoff (Fig. 11a). Local RRH and storage through local wells, ponds and sand dams increased the simulated baseflow by 5, 2 and 1%, respectively (Fig. 10), thereby increasing groundwater discharge to the nearby wetlands, rivers and drainage network, thus improving ecological sustainability.

On the other hand, surface runoff was reduced by up to 27, 11 and 1%, respectively, due to local RRH and storage through small-scale water wells, ponds and sand dams recharge (Fig. 11a). This reduction in surface runoff could also decrease the risk of floods and erosion.

#### Impact of land use–land cover changes

*Impact of deforestation and afforestation:* Scenario simulation results showed that deforestation could lead to an increase in groundwater recharge across the LRB, while, conversely, afforestation could potentially decrease it (Fig. 11b). In the case of deforestation, the reduction in vegetation cover was assumed to allow more rainfall to reach the ground, enhancing groundwater recharge. Conversely, afforestation was assumed to increased canopy interception and transpiration, limiting the amount of water reaching the ground and potentially reducing groundwater recharge.

In the deforestation scenario, groundwater recharge could increase by up to 20%, with an average increase of 12%, while average runoff would increase by 5%. In the afforestation scenario, groundwater recharge could decrease by up to 42%, with an average decrease of 30%, while average runoff would decrease by 11% (Fig. 11b). Increased runoff due to deforestation, however, would increase floods and erosion. Conversely, a decrease in runoff due to afforestation would decrease floods and erosion.

Increased groundwater recharge resulting from deforestation could lead to a rise in groundwater levels by up to 10 to 15 m in the deforested areas (Fig. 9). The impact is shown to be relatively greater around the Mahalapswe, Lotsane and Motloutse sub-watersheds in Botswana; the Lower Olifants and Letaba subwatersheds in South Africa; and the Mwenezi and Bubi sub-watersheds in Zimbabwe (Fig. 9 and Mosase and Ahiablame 2018). This is because most of the forested areas in the basin lie within these regions of these countries. Consequently, deforestation would have a more significant impact in these areas. Conversely, reduced groundwater recharge would contribute to a lowering of groundwater levels by up to 15 m in the afforested areas (Fig. 9). Although the afforestation impact is relatively greater in the three upstream countries compared to Mozambique, the most impacted areas lie in Zimbabwe and along the border between South Africa and Mozambique. The afforestation scenario decreased simulated baseflow by up to 2%, while deforestation increased it by up to 1.5% (Fig. 10).

### Comparison between scenarios

Overall, results of the modelling scenarios suggest that increased groundwater abstraction and afforestation will have a negative impact on groundwater recharge (decrease), level (decrease) and baseflow (decrease), thereby reducing groundwater storage, increasing the likelihood of groundwater drought occurrences and increasing vulnerability to droughts. This decrease in baseflow can have significant impacts on river flow and wetlands, not only locally but also in neighbouring (especially downstream) regions. Areas with shallow water tables that experience a substantial decline of the water table are likely to experience a decline of shallow surface moisture conditions and therefore become more vulnerable to both floods and droughts.

On the other hand, simulations showed that the various scenarios of MAR could all have a positive impact on groundwater recharge (increase), level (increase), baseflow (increase) and surface runoff (decrease), thereby reducing impacts and increasing resilience to drought and enhancing groundwater sustainability throughout the basin. In MAR scenarios, capturing and storing the excess floodwater during flooding events could reduce the impact of extreme floods and increase groundwater storage. This additional storage can serve as an essential source of water during droughts and support river baseflow, thereby reducing the impact of droughts. In this way, resilience to hydrological extremes in the LRB can be improved.

While deforestation could potentially increase groundwater recharge (Fig. 9) and enhance resilience to droughts, it may also lead to increased surface runoff (Fig. 11), increasing the likelihood of floods. On the other hand, although afforestation could decrease groundwater recharge (Fig. 9), resulting in reduced groundwater availability and diminished resilience to drought, it may also decrease surface runoff (Fig. 11), thereby reducing the occurrence of floods and lessening vulnerability to flooding.

Comparing the results of MAR scenarios (Figs. 9, 10 and 11) suggest that MAR utilising RRH through local wells could yield the most substantial increase in groundwater storage across the basin, while also significantly boosting groundwater recharge and reducing surface runoff. It is worth noting that if applied to all dams across the LRB, MAR using injection wells may have a greater impact than local wells. However, it is also subject to greater expenses. Taking all aspects into account, MAR using local wells potentially offers the most effective solution among the considered scenarios for reducing impacts and enhancing basin resilience to flood–drought cycles, promoting groundwater sustainability and fostering ecological sustainability. This also aligns with stakeholder perceptions and preferences on the effectiveness and feasibility of implementing measures for resilience and sustainability. However, local well injection and local pond infiltration may mobilise contaminants affecting groundwater quality, unlike sand dams.

However, achieving the maximum benefit of RRH through local wells may not be possible, especially during extreme rainfall events if the rainfall exceeds the potential capacity of the RRH through local wells. In that case, a combination of RRH and storage through local wells, along with floodwater capture and infiltration in small water reservoirs and sand dams, could be very effective and more beneficial.

So, at the local level, the best solution to building resilience against these hydrological challenges (floods, droughts, groundwater depletions) would involve a combination of different strategies. The best approach could be a combination of afforestation, MAR and maintaining increased abstraction at a sustainable level. Spatial variability should be taken into account during the implementation of the measures. For example, afforestation should be focused on flood-prone areas characterised by steep slopes and less permeable surfaces where it could decrease runoff and erosion, rather than on non-flood-prone areas where it may decrease groundwater recharge and thereby reduce groundwater storage. MAR using injection wells should not be implemented very close to the river, especially in aquifers characterised by productive sediment and shallow groundwater levels. Geology should be accounted for during the implementation of MAR. Stakeholders’ preferences, their livelihoods and traditional practices should also be considered during the implementation of the management measures.

## Discussion

### Model performance and groundwater flow dynamics under data paucity

The availability of sufficient data, and the accuracy, spatial and temporal resolution of these limited available data were major challenges in this study. Basin-scale detailed geological information, crucial for developing a basin-scale hydrogeological model, was not readily available. While regional surface geological data (geological maps) were adequate at the regional scale, the availability of vertical stratigraphy or lithological data is limited. Additionally, continuous hydrogeological data, spatio-temporal groundwater monitoring data (e.g. groundwater levels), aquifer properties data, well characteristics (depth, abstraction rates, etc.) and river physical properties (river water level, river bottom level, riverbed thickness, riverbed hydraulic conductance) are lacking.

Spatio-temporal time series of groundwater abstraction data were not available. However, a representative groundwater abstraction dataset based on borehole yield, license rate and population growth rate, was created with the guidance of local stakeholders.

Although limited data availability was an issue, the model’s acceptable simulation capacity was confirmed through the comparison of observed and simulated groundwater levels, depths and statistical indicators (Fig. 4 and Table 2). The model successfully captured the yearly and seasonal variations and trend of groundwater depth between 2009 and 2019. Despite the limitations in the availability of good-quality data, the model conceptualisation and related assumptions were thoroughly discussed with local experts. Ritzema et al. (2010) also reported that local expert knowledge can enrich the utility of simulation models by bridging the gap caused by the absence of long-term data records. Integrating stakeholders’ location-specific knowledge with explicit scientific expertise can significantly enhance the accuracy and applicability of the models.

Model calibration also confirmed the widely accepted conceptual model of basement aquifers with greater values of hydraulic conductivities and lesser specific storage in the fractured zone (saprock) compared to overlying weathered basement (saprolite), indicating a greater groundwater flow capacity of fractured basement than weathered basement. The area with dykes and faults was calibrated with greater hydraulic conductivities than the surrounding basement rocks, consolidated sedimentary and volcanic rocks. The calibrated values are within the range of measured and estimated hydraulic properties values already published in scientific journals from the same area (Table 1 and S4 of the ESM).

However, several modelling choices—including the assumption of a uniform aquifer thickness, the 1000 m × 1000 m spatial resolution and the representation of boundary conditions and structural controls—were necessary due to data limitations, the study’s specific objectives and scale, and the need to maintain computational efficiency for such a large basin-scale model. While acceptable for this study’s objectives and scale, these simplifications have implications for interpretations at the local level (de Vries 2025). Uncertainty analyses by de Vries (2025), summarised in section “Model performance and groundwater flow dynamics under data paucity” of the ESM, suggest that while groundwater levels and surface water–groundwater interactions exhibit considerable uncertainty at the local level, broader basin-scale trends and river-aquifer interactions are preserved and do not fundamentally alter the main findings. However, for local-level applications, the associated uncertainty should be addressed in future research.

While spatially distributed groundwater recharge has been simulated, the WetSpass model does not include a routing process through the unsaturated zone, introducing uncertainty in the simulated recharge. This issue and its impact on uncertainty need to be investigated in future studies.

### Identification of management scenarios and their impact evaluation

In the MCGM approach, stakeholders were involved at every step, to identify seven management scenarios. This collaborative modelling approach has demonstrated that it helps to improve understanding, to parameterise and to verify complex hydrogeological models with reliable acceptable accuracy, even in data-scarce transboundary basins. Basco-Carrera et al. (2017) highlighted that participatory modelling involves a lower level of participation from key stakeholders than collaborative modelling and emphasised the importance of active participation of a variety of stakeholders rather than specifically targeting certain stakeholders, similar to some existing participatory modelling approaches. Elshall et al. (2020) also highlighted the importance of involving a broader range of relevant stakeholders throughout the development process. White et al. (2010) and Heink et al. (2015) found that stakeholder involvement can lead to better informed and more sustainable management decisions. Custodio et al. (2019) also found that stakeholder consensus can lead to better results.

This iterative engagement with stakeholders increased their confidence and trust in the model itself, in the identified scenarios and results, and in the policy recommendations drawn. This comprehensive involvement also ensures that the management scenarios are well-informed and relevant to the needs and aspirations of the local community and other stakeholders in the basin, along with creating a sense of ownership of the results by them.

Results show that increased groundwater abstraction and afforestation could significantly decrease the groundwater level and increase the likelihood of groundwater drought occurrences reducing the resilience to drought. This reduced groundwater level may adversely affect crop production as it reduces the available soil moisture necessary for optimal crop growth. The reduction in groundwater level may also adversely impact ecological sustainability, as it would decrease groundwater discharge to wetlands and rivers. This impact could be even greater due to increasing drought frequency and intensity in the Limpopo River basin (Mupangwa et al. 2021).

The impact of increased abstraction is relatively low in Botswana, Zimbabwe and Mozambique compared to South Africa due to lesser population density and associated lesser groundwater abstraction. This suggests that there may be more room for further groundwater development in these three countries. However, the lack of groundwater abstraction data from these three countries compared to South Africa implies these inferences from the model should be taken cautiously and more information sought on groundwater use. Model simulations illustrate the great risk of water resource depletion posed by further development of groundwater resources in the more impacted areas, especially in the already stressed sub-catchments in South Africa. Reducing the groundwater abstraction to the sustainable limit in those areas and/or jointly implementing MAR is recommended. Further studies on the identification of sustainable abstraction level are important. Although the impact is limited, greater abstraction in South Africa still affects neighbouring countries. Maass-Morales et al. (2024) also highlighted that the impact of anthropogenic activities (e.g. groundwater abstraction, land use and land cover changes) can be felt in the neighbouring countries within a TBA.

Simulation results show that afforestation increases interception and transpiration, reducing groundwater recharge and runoff. Conversely, deforestation would reduce interception and transpiration while increasing groundwater recharge and runoff. Deforestation has been shown in several studies to reduce ET, potentially increasing groundwater recharge and water availability (Woodward et al. 2014; Ouyang et al. 2019). On the contrary, Bala et al. (2007) reported results contradicting the idea that deforestation reduces ET and increases water availability, noting that large-scale deforestation has a net cooling influence. However, the processes involved are complex and vary depending on climate, vegetation, local conditions, slopes, land cover and the type of deforestation. The specific circumstances under which deforestation might increase recharge remain unclear. Therefore, deforestation should be approached with caution as a management strategy; instead, afforestation should be managed appropriately. Many researchers have suggested that afforestation can increase evaporation and decrease local water availability (Farley et al. 2005; Jackson et al. 2005; Filoso et al. 2017). Hoek van Dijke et al. (2022) found that global afforestation could lead to a decrease in water availability of up to 38% in regions across the world. Simulation results also show that deforestation would increase floods and erosion due to increased runoff, while afforestation decreases them by reducing runoff. Bradshaw et al. (2007) and Peptenatu et al. (2020) also demonstrate that deforestation can significantly increase the risk of flooding.

Afforestation might also increase the surface runoff time of precipitation, thereby enhancing infiltration. However, this effect was not considered in this study and requires further research.

The results show that all the MAR options considered enhance groundwater storage, thereby improving groundwater sustainability and reducing groundwater drought, resulting in increased resilience to drought. The findings also indicate that MAR can enhance baseflow, leading to greater water availability for surface water, wetlands and rivers. This additional water to the rivers may provide a solution to surface water scarcity and contribute to ecological sustainability, especially during extreme drought periods. Additionally, MAR is found to reduce surface runoff, potentially mitigating flooding and land subsidence risks, further enhancing flood resilience. The model demonstrates that MAR has the capability to capture excess floodwater and store it underground, thus boosting groundwater storage and availability during droughts. Through this integrated approach, MAR can enhance resilience to hydrological extremes, such as floods and droughts.

Recently published papers show that MAR is increasingly being considered worldwide as a potential solution for aquifer sustainability and improving resilience to hydrological extremes (Maliva 2020; Zhang et al. 2015; Fakhreddine et al. 2021; Levintal et al. 2023). MAR has been applied and demonstrated for various purposes, including for drinking water supply, mitigating land subsidence, increasing resilience to hydrological extremes and ecosystem restoration. Sikka et al. (2021) and Scanlon et al. (2023) argue that MAR is crucial for sustainable water management and to address water scarcity. MAR can help manage excess surface water, reduce the negative impact of flooding and provide water storage for dry seasons. Alam et al. (2020) and Wendt et al. (2021) demonstrated that MAR could mitigate the impact of increased groundwater abstraction in Central Valley in California, USA. He et al. (2021) and Dillon et al. (2019) also highlighted that MAR is a potential solution for integrated management and increasing resilience to droughts, floods and depleted groundwater resources.

While the impact of injection wells on groundwater levels at the basin scale is less than local wells and ponds, their influence on baseflow is more significant, almost matching that of local wells. This is because injection wells are installed near rivers, utilising excess water from dams during high water levels and flooding events. Consequently, more water quickly flows back into the river rather than increasing groundwater levels. This study primarily focused on the source of water and stakeholder preferences to mitigate the negative impacts of floods. Future research should also consider factors such as geology, aquifer storage capacity and distance from the river.

Modelling results, aligning with stakeholders’ preferences, suggest that MAR using local wells is the most effective solution at the basin level, with the most impact on South Africa and Zimbabwe, followed by Botswana, enhancing resilience to hydrological extremes. This shows that collaborative modelling can develop effective strategies to enhance resilience to hydrological extremes and meet stakeholder needs.

Having all these benefits, the implementation of MAR is not very straightforward and needs to consider some physical, policy and social aspects. Possible negative impacts on water quality, expenses, possible health hazards and willingness of the local people and policymakers need to be accounted for. The availability of required water for MAR also needs to be accounted for (He et al. 2021). The source of water and the approach to MAR have a strong bearing on the risks of contamination. MAR water can be easily and safely used for irrigation purposes, but using it for drinking purposes requires extra attention regarding water quality, which is dictated by the source of water used for MAR, the methods used for MAR and how long water has been in the subsurface before using it. For example, water from MAR using rainwater harvesting through local wells can be used for both drinking and irrigation as the quality of the source water (rainwater) is considered to be good. On the other hand, water from MAR using small water reservoirs (e.g. sand dams) depends on the quality of the reservoir water. The implementation of MAR through local wells in South Africa is promising as it has been explicitly identified as a preferred approach by stakeholders along with local ponds, whereas stakeholders from Botswana and Zimbabwe preferred the use of sand dams. MAR through local wells and local ponds was also preferred by stakeholders from Botswana. For details regarding stakeholder preferences, see Van Loon et al. (2025).

In the LRB, MAR could be a potential solution for water sustainability, as stakeholders highlighted that groundwater suitability varies by usage (drinking, irrigation), noting that transboundary mining and excessive agricultural abstraction actively reduce the effective benefits of long-term water management plans (Van Loon et al. 2025). LRB communities are already facing water quality issues. Geris et al. (2022) reported that a prior drought followed by a sudden flood (e.g. the 2017 event) can cause significant water contamination. They also highlighted that upstream mining operations (primarily in South Africa) heavily degrade and alter downstream water quality. Van Loon et al. (2025) reported that community focus groups explicitly noted severe water quality degradation during droughts. Livestock farmers from South Africa highlighted that when groundwater supplies dry up, the water tastes salty and brackish. However, because they lack alternative resources, they are forced to keep using it to cook food and sustain their livestock. In such circumstances, the potential for MAR to provide a solution to improve water quality needs to be investigated in future research, with attention paid to societal practices.

### Stakeholder participation and challenges

Whilst initial institutional stakeholder mapping was straightforward, engaging government officials in the modelling workshops became extremely difficult following the onset of the COVID-19 pandemic due to widespread office closures and emergency disaster management duties. Nevertheless, the iterative national and transboundary workshops were effective for the collaborative development of management scenarios; participating stakeholders actively identified vulnerable areas and ranked developed strategies by feasibility. While researchers made every effort to keep stakeholders closely engaged at every step of the modelling process, the level of enthusiasm and participation among all stakeholders varied between the different steps. Local community representatives, extension officers and institutional stakeholders whose backgrounds were not hydrology were relatively more enthusiastic to hear and discuss scenario results but were less engaged during the conceptual model development and scenario definition. In contrast, local water experts were equally enthusiastic about both conceptual model development and scenario co-creation. Importantly, the collaboration revealed the high potential for acceptance of local solutions, including low-cost, decentralised community strategies, which were perceived as promising for enhancing local livelihoods and food security without state bureaucracy.

Unlike upstream community focus group discussions which were held in local languages of the participants across the four basin countries, the stakeholder modelling workshops (including scenario co-creation) were held in English with Portuguese translation, and included local community members who could communicate in these languages, alongside extension officers, institutional stakeholders, national water agencies and ministry officials. This is a notable limitation to the inclusiveness of the collaborative modelling approach which should be addressed in future research. Additionally, to minimise the impact of terminology misunderstandings during workshops, the conversation started with a very simple and understandable presentation, featuring clear explanations of the terminology and clarification of the concepts and assumptions used in the modelling. Van Loon et al. (2025) also highlighted from the same study that highly technical forecast metrics were identified as a major barrier to community adoption. To bridge this gap, information flows were mediated by local agricultural extension officers, who translated scientific data into actionable practices for local farmers. Further details on these additional efforts can be found in Van Loon et al. (2025).

## Conclusions

An iterative, multisector collaborative modelling and knowledge co-creation approach was developed in the relatively data-scarce LRB, which is greatly vulnerable to hydrological extremes. Through active involvement of stakeholders at different levels, the participatory modelling results led to identification and co-design of water management solutions potentially effective in building resilience to hydrological extremes.

The comparison of observed and simulated groundwater levels, depths and all statistical indicators (e.g. NSE 0.98, and KGE 0.95) confirmed the acceptable simulation capacity of the model. This demonstrates that it is possible to develop a regional-scale integrated hydrogeological model with acceptable accuracy even in a data-scarce, complex transboundary basin using a collaborative framework.

Through iterative collaborative modelling and knowledge co-creation, identified seven management scenarios were evaluated using the calibrated model. Analysis of identified management scenario results suggest that MAR using injection wells next to large dams, RRH through local wells, RRH through local ponds and sand dams can reduce the risk and impact of floods and droughts at the basin scale by increasing groundwater recharge, levels, baseflow and decreasing runoff, thereby increasing resilience to these hydrological challenges. However, increased groundwater abstraction may lead to a decrease in groundwater level, by up to 25 m. Afforestation will decrease groundwater storage enhancing drought, but also decrease runoff, reducing floods and erosion. Deforestation could also adversely increase runoff. Both afforestation and deforestation should be managed appropriately and with particular attention to the local context.

The simulated results also show that MAR using RRH through small-scale water well recharge is the most effective strategy in reducing the risk and impact of floods and drought at the basin scale in the LRB. A combination of different strategies at the local level would be the best solution to building resilience against these hydrological extremes. A combination of afforestation to reduce runoff, MAR to increase groundwater storage, and limiting the increase of groundwater abstraction could be beneficial. However, the combination of measures will depend on local topography, geology, groundwater storage, stakeholders’ preferences, livelihoods and traditional practices, which should be considered before implementation. For example, afforestation should target flood-prone steep-slope areas, and avoid non-flood-prone areas where it may result in a decrease of groundwater recharge, storage and river baseflow, while MAR should avoid riverside areas with productive sediment and shallow groundwater. Local solutions such as MAR using RRH through small-scale wells and ponds should be promoted in the upstream and central parts of the basin where groundwater depth is high. Additionally, the potential risk of groundwater contamination from MAR should be considered. Groundwater abstraction should be kept sustainable across all countries, especially in South Africa. The quantitative results and management recommendations from this study will aid stakeholders and policymakers in making informed decisions for their areas and at the basin level to enhance resilience to hydrological extremes and improve groundwater sustainability. This includes decisions on licensing groundwater abstraction wells, MAR and afforestation or deforestation, which they have been seeking as expressed in stakeholder workshops and discussions.

## Supplementary Information

Below is the link to the electronic supplementary material.

Supplementary file1 (PDF 844 KB)

## Data Availability

The hydrometeorological and hydrogeological data used in this paper are summarised in the tables, figures, and references, with links provided where necessary and accessible. The models used for this study (MODFLOW and WetSpass) are open-source and freely accessible. Model input and output data will soon be available (currently being uploaded) via the UK Environmental Information Data Centre: Mustafa and Comte (2026). Basin-scale hydrological modelling data of the Limpopo River Basin, southern Africa. https://catalogue.ceh.ac.uk/documents/cb12bb29-5639-4788-bfbc-249537f38d37. The data is also available from the authors upon request (syed.mustafa@wur.nl).
