# Supplementary material for: Groundwater–surface water interactions and resilience to hydrological extremes in a data-sparse arid transboundary river basin: Insights from collaborative groundwater modelling
Source: Hydrogeol J. 2026 Aug 14;34(6):1817–46. doi: 10.1007/s10040-026-03143-x (PMC13562358; doi:10.1007/s10040-026-03143-x)
Supplement: Supplementary file 1 [file 10040_2026_3143_MOESM1_ESM.pdf]

Hydrogeology Journal: Electronic supplementary material

Groundwater-surface water interactions and resilience to hydrological extremes in a data-sparse arid transboundary river basin: Insights from collaborative groundwater modelling

Syed Md Touhidul Mustafa [a, b, *], Jean-Christophe Comte [a, *], Fulvio Franchi [c], Alessia Matano [d], Anne F. Van Loon [d], Sithabile Tirivarombo [e], Luis Artur [f], Zareen Bharucha [g], Annatoria Chinyama [h], Farisse Chirindja [f], Rosie Day [i], Joyce Dube [j], Stephen Hussey [j], Edward Nesamvuni [k], Wandile Nomquphu [l], Oluwaseun Olabode [a], Melanie Rohse [g], Simon Taylor [g], Jarmo de Vries [b]

[a] School of Geosciences, University of Aberdeen, Aberdeen, UK

[b] Now at Hydrology and Environmental Hydraulics Group, Wageningen University & Research, Wageningen, the Netherlands

[c] Dipartimento di Scienze della Terra e Geoambientali, Università degli studi di Bari – Aldo Moro, Bari, Italy

[d] Institute for Environmental Studies, Vrije Universiteit Amsterdam, Amsterdam, The Netherlands

[e] Department of Earth and Environmental Science, Botswana International University of Science and Technology (BIUST), Palapye, Botswana

[f] Eduardo Mondlane University, Maputo, Mozambique

[g] Anglia Ruskin University, Cambridge, UK

[h] National University of Science and Technology, Bulawayo, Zimbabwe

[i] School of Geography, Earth and Environmental Sciences, University of Birmingham, Birmingham, University of Birmingham, Birmingham, UK

[j] Dabane Trust, Bulawayo, Zimbabwe

[k] Department of Sustainable Food Systems and Development, University of Free State, Bloemfontein, South Africa

[l] Water Research Commission, Pretoria, South Africa

* Corresponding authors: Syed M. T. Mustafa, E-mail: syed.mustafa@wur.nl; Jean-Christophe Comte, E-mail: jc.comte@abdn.ac.uk

ESM 2.1 Multisector Collaborative Groundwater Modelling approach

Table S1: Summary of stakeholder workshops and group discussions including timing and participant types and numbers

Time	Workshop/discussion	Participant numbers	Stakeholders
20 September 2020	Group discussions	10	Project partners and their representatives from all four countries, including university professors (from University of Free State, in South Africa, Botswana International University of Science & Technology, National University of Science and Technology in Zimbabwe, and Eduardo Mondlane University in Mozambique), MS students, representatives of NGOs (e.g, Dabane Trust in Zimbabwe, German Red Cross in Mozambique), and research organizations
18 December 2020	Discussion sessions	17	Key stakeholders from national water resource management institutes identified via snowball sampling include ARA-Sul (Mozambique), Directorate of Water Supply and Sanitation in Mozambique, Zimbabwe National Water Authority (ZINWA), Ministry of Energy, Water & Climate in Zimbabwe, Water Utilities Corporation (WUC: Botswana), Department of Water Affairs in Botswana, Department of Water and Sanitation in Botswana, and Water Research Commission, and Department of Water and Sanitation (South Africa)
02-03 June 2021	Transboundary workshop (Two-days)	35	Stakeholders were again selected through snowball sampling, representing locally active international research institutes such as IWMI: International Water Management Institute, national water management institutes (participated in institutional discussions), and transboundary institutes like SADC: Southern

			African Development Community, SADC-GMI, and LIMCOM: Limpopo Watercourse Commission
April and May 2022	4 national workshops (one day each)	Botswana: 10<br>South Africa: 5<br>Zimbabwe: 5	Representatives of national water institutes, extension officers, and policymakers (e.g., representatives from the different Ministries)

ESM 2.2 Model input and data sources

Table S2: Data used in this study including their sources.

**Data**	**Source**	**Spatial resolution***	**Temporal resolution**	**Processing tool/method**
Digital elevation model (DEM)	Shuttle Radar Topography Mission (SRTM)<br>https://earthexplorer.usgs.gov/	30 m × 30 m	Static raster map	ArcGIS
Land-cover map	European Space Agency GlobCover Portal<br>http://due.esrin.esa.int/page_globcover.php<br>Sentinel-2A (European Space Agency satellite) data	20 m × 20 m	Composite map for<br>2009 and 2016 (2016 is used)	ArcGIS<br>Lookup according to the WetSpass model
Soil map	International Soil Reference and Information Centre (ISRIC) available in Hengl et al. (2017): https://www.isric.org/index.php/	250 m × 250 m	Static raster map<br>Texture class (USDA system) at seven standard depths	ArcGIS<br>Lookup according to the WetSpass model

Slope map	Calculated from DEM	30 m × 30 m	Static raster map	ArcGIS
Precipitation	Climate Hazards Group InfraRed Precipitation with Station (CHIRPS) data from<br>https://www.chc.ucsb.edu/data	5.5 km × 5.5 km	Daily raster map from 1981 to 2020	Python & ArcGIS (Monthly sum is used in WetSpass)
Evapotranspiration (ET)	The actual EvapoTranspiration and Interception (ETI) products from FAO Water Productivity Open-access portal (WaPOR):<br>https://wapor.apps.fao.org/home/WAPOR_2/1	250 m × 250 m	Monthly raster map from 2009 to 2020	Python & ArcGIS (Monthly sum is used in WetSpass)
Temperature and Wind speed	Climate Forecast System Reanalysis (CFSR) data from Texas A&M university spatial sciences: https://globalweather.tamu.edu/	38 km × 38 km	Daily grided data	Python & ArcGIS (Monthly average is used in WetSpass)
River discharge	Global Runoff Data Centre (GRDC)<br>https://www.bafg.de/GRDC/EN/Home/homepage_node.html	Observed data of the 65 stations within LRB	Varies with measuring station	Python & ArcGIS
River network	Global Runoff Data Centre (GRDC)		Static shapefile	ArcGIS
River water level	Copernicus Global Land service, hosted by VITO on behalf of the European Commission Joint Research Centre (JRC)<br>https://land.copernicus.eu/global/products/wl	Observed data of the 35 stations within LRB	Static water level	

DAM location and outflow	SADC Groundwater Information Portal (SADC-GIP), SADC-GMI<br>https://sadc-gip.org/; SADC: Southern African Development Community			Python
Groundwater level (static)	SADC Groundwater Information Portal (SADC-GIP), SADC-GMI<br>https://sadc-gip.org/	Observed water level of 12247 boreholes across the LRB	Static water level	ArcGIS
Groundwater level (transient)	SADC Groundwater Information Portal (SADC-GIP), SADC-GMI<br>https://sadc-gip.org/	Observed water level of 250 boreholes across the LRB	Monthly water level for the period 2009 – 2019	Monthly average groundwater depth is calculated and used in WetSpass
Hydraulic conductivity and other hydraulic properties	Based on in-situ measurements across the LRB collected from different published articles (Table ESM4).			
Geological information, including surface geology, depth to bedrock, dykes, faults and	1a. Africa Groundwater Atlas hydrogeology maps: Botswana, Mozambique, Zimbabwe.<br>http://earthwise.bgs.ac.uk/index.php/Hydrogeology_by_country<br>1b. Council for geoscience: South Africa	-	Static shapefile	Python & ArcGIS

geological Cross-section.	http://www.geoscience.org.za/index.php<br>2. U.S. Geological Survey: for whole LRB<br>https://certmapper.cr.usgs.gov/data/apps/world-maps/<br>3. SADC Groundwater Information Portal (SADC-GIP), SADC-GMI<br>https://sadc-gip.org/			
Groundwater abstraction: borehole yield and licensed rates	SADC Groundwater Information Portal (SADC-GIP), SADC-GMI<br>https://sadc-gip.org/	-	Static shapefile	Python & ArcGIS
Population: density and change rate	https://sedac.ciesin.columbia.edu/data/sets/browse?facets=theme:population&facets=data-type:raster		Raster map from 1981 to 2050	Python & ArcGIS
Irrigated or rainfed crops, the types of crops and the water requirements.	https://www.uni-frankfurt.de/45218031/data_download	-	Static shapefile	ArcGIS
Historical floods and drought	Collected from Lindevall, (2017) and adopted through stakeholders workshops			

events and the extent	

* Spatial resolution of all maps is converted to 1000 m x 1000 m to use in our model.

ESM 2.3 Model setup

Table S3: MODFLOW-2005 packages used in this study

Abbreviation	Model package name and type	To represent and simulate
Groundwater Flow process:		
DIS	Discretization package	Spatial, temporal, and vertical discretization: including mesh size and layer property
BAS	Basic Package	Model domain, boundary conditions, starting heads, constant heads, and IBOUND arrays: the locations of active, inactive, and specified head cells (Specified Head Boundaries)
Groundwater flow packages:		
LPF	Layer-Property Flow Package	Aquifer properties: including horizontal conductivity, vertical conductivity, specific storage, specific yield and aquifer types: confined, and confined/unconfined convertible<br>The fault/fracture has also been represented within this package using higher hydraulic conductivities.
Boundary Condition Packages:		

RCH	Recharge Package (Specified Flux Boundaries)	To implement spatially distributed groundwater recharge; to implement the MAR using rainwater and runoff harvesting (RRH) through small-scale water reservoirs/local ponds, local wells and sand dams; and also to implement the impact of land use-land cover changes
WEL	Well Package (Specified Flux Boundaries)	To implement groundwater abstraction across the LRB and evaluate its impact; also, implement the MAR using injection wells next to large dams
DRN	Drain Package (Head-Dependent Flux Boundary)	To implement smaller ephemeral distributaries and tributaries across the LRB
RIV	River Package (Head-Dependent Flux Boundary)	To implement the major, mostly perennial rivers across the LRB and to simulate river-aquifer interaction
Observation Process		
HOB	Head-Observation Package	For model calibration and validation using observed groundwater level data
Solvers		
PCG	Preconditioned Conjugate Gradient Solver with Improved Nonlinear Control	Solver input criteria
Output Control		
OC	Output Control	Specify when simulated heads and water budget are printed in the listing file or saved to an external file

ESM 2.4 Model parameterization

Table S4: Hydraulic conductivities for different geological environments across the LRB and similar geological environment across the world

Geological environment	Materials	Hydraulic Conductivity (m/d)	Study area	Method	Sources
Basement	Crystalline bedrock	0.71 (0.51-0.91)	Limpopo Province, South Africa (Limpopo Plateau and Letaba Lowveld	Pumping test (summary of 2,500 test)	Holland and Witthüser (2011)
	Crystalline bedrock	0.1-1	Limpopo Province, South Africa	Pumping test	Holland, 2011
	Basement aquifers	0.2-2.5	Limpopo Province, South Africa	Constant discharge and step drawdown pumping tests (summary of 1000 test)	Dippenaar et al., 2009
	Crystalline bedrock aquifers	0.24	Seeta, Mubende, and Rukungiri in Uganda	Pumping tests	Maurice et al., 2019
	Fractured hard-rock aquifers	0.69 (0.27 – 1.1)	South Africa		Holland, 2012

	Basement Complex (granite and gneiss)	0.25 (0.05 – 0.5)	Zimbabwe		Upton et al., 2017; BGS, 2019
	Archean basement	0.098 – 0.488	Botswana	Pumping test	Marema et al., 2023
	Hard-rock semi-arid environment	0.41: weathered basement, and<br>0.86 Fractured aquifer	Hout<br>catchment, Limpopo Province, South Africa	Modelling	Ebrahim et al. (2019)
	Fractured basalt rock	0.003	Stormberg Basalt Aquitard (SBA), Botswana	Pumping tests	Brassington, 1998; Snowy Mountains Engineering Corporation and EHES Consulting Engineers, 2006; discussed and cited in Lekula et al. 2018
	Hard-rock aquifer	0.38	Maheshwaram watershed, India	Slug tests, injection tests, flowmeter tests, and pumping tests	Maréchal et al. 2004
	Igneous – Karoo Basalt	0.26 – 2.57	Zimbabwe	Pumping tests	Upton et al., 2017; BGS, 2019

Volcanic and sedimentary rocks	Volcanic rocks	0.5	Botswana		United Nations, 1989
	Upper Karoo sandstone	0.05 – 5.18	Mid-Zambezi basin, Zimbabwe	Pumping tests, Sieving analyses	Larsen et al., 2002; Beasley, 1983
	Tuli syncline Basalts	0.003 – 1.6	Botswana		Snowy Mountains Engineering Corporation and EHES Consulting Engineers, 2006
	Fractured granite aquifer	3.0-5.0	Dulapally Basin, Hyderabad, India	Pumping test	Rushton and Weller (1985)
Consolidated materials	Consolidated field below volcanic formation	0.1 – 2.09	Bulawayo aquifer, Zimbabwe	Pumping test	Rusinga & Taigbenu, 2005
	Ramotswa Dolomite	2.69 – 5.91	Botswana	Pumping test	DWA, 1991
	Sandstones	0.1-10	Lebung aquifer, Botswana	Pumping tests (from 358 test)	Brassington, 1998; Lekula et al. 2018
	Taupone Dolomite	0.89 – 1.33	Botswana/South Africa	Pumping test	Snowy Mountains Engineering Corporation and EHES Consulting Engineers, 2006
	Dolomite	4.92 – 11.7	Botswana	Pumping test	Staudt, 2003

Consol. Intergranular sediments	Unconfined Kalahari Sand Unit (KSU)	1.0 – 15.0 (Horizontal)	Kalahari Sand Unit (KSU), Botswana	Pumping tests	Snowy Mountains Engineering Corporation and EHES Consulting Engineers, 2006; discussed and cited in Lekula et al. 2018
Unconsolidat edsediments/ poorly consolidated aquifer*	Sand River Deposits	33 – 284	Limpopo River, Mozambique<br>Chókwè+Guijá	Stable isotope analysis, sediment classification	Saveca et al., 2022
	Alluvial	32 - 639	Nhandugue River, Mozambique	Grain size analysis	Arvidsson et al., 2011
	Sand	96–100	Shashani, Zimbabwe	Grain size analysis	Hussey, 2003
	Alluvial deposit	26.8<br>61.3<br>62.8	Mzingwane Catchment, Zimbabwe	Slug test<br>Permeameter test	Love et al., 2007
	Alluvial sands	98 (14 – 181)	Shashane, Huwana, Wenlock, and Dongamuzi catchments in southern Zimbabwe	Constant head permeameter	Mansell and Hussey, 2005
	Alluvial aquifers	40 - 200	Mzingwane catchment, Zimbabwe	Grain size analysis	Moyce et al., 2006

	Sand Rivers deposits	41 - 98	Shashani, Zimbabwe	-	Blok, 2017
	Alluvial aquifers	60	Mzingwane river in the LRB, Zimbabwe	Grain size analysis/Sieve analysis, pumping tests, permeameter	Masvopo, 2008
	Sand rivers	32–46	Mzingwane River in LRB, Zimbabwe	Laboratory test	Love et al., 2011
	Alluvial aquifer	69	Mnyabezi, southern Zimbabwe	-	de Hamer et al., 2008
	Alluvial aquifers	20 – 60	Runde & Sessami Rivers in Zimbabwe	Grain size analysis	Owen, 1989
	Sand river deposits	700	Tati River, Botswana	-	Herbert et al., 1997
	Sand rivers	80 (20 – 250)	Mahalapshwe River, Botswana	Grain size analysis	Thomas and Hyde, 1972
	Sand	260	Sand Rivers in Botswana	Pumping test	Nord, 1985
	Sand	20 – 70	Mara Basin, Kenya	Probing and Electrical Resistivity Tomography techniques	Wekesa et al., 2020

	Sand river deposits	120	Limpopo River at Messina, South Africa	-	Mulder, 1973
	Alluvial aquifer	170 – 290<br>118 (20 – 330)<br>71 (10 – 260)	Molototsi sand river, Limpopo, South Africa	Pumping/recovery<br>Test, Falling head permeameter, HydrogeoSieveXL based on grain<br>size analysis	Walker et al., 2018

* Check Walker et al. (2018) for more detailed information on hydraulic conductivities and thickness of unconsolidated alluvial aquifer.

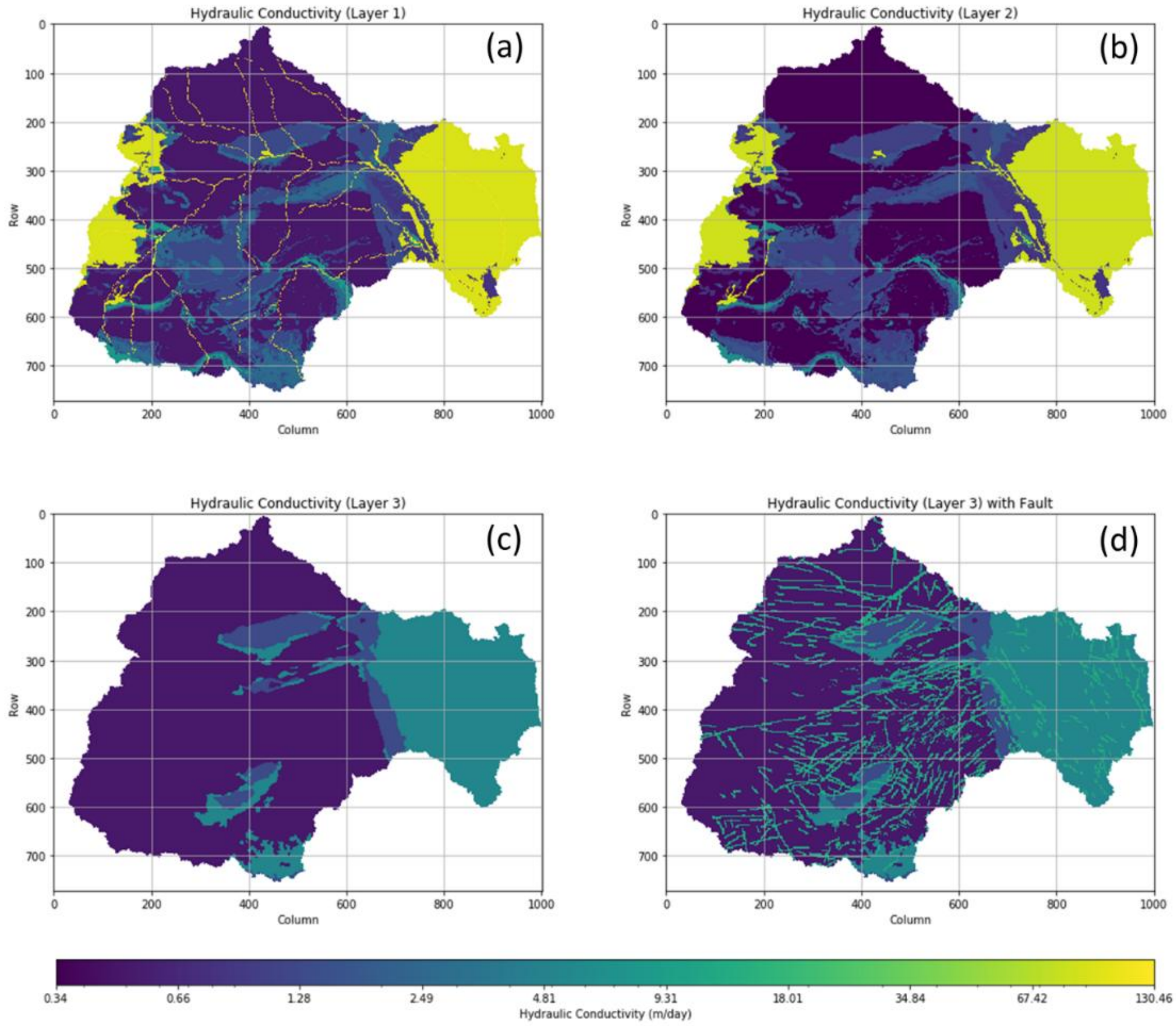

Figure S1. Horizontal hydraulic conductivities for different geological environments and layers: (a) Layer 1, (b) Layer 2, (c) Layer 3, and (d) Layer 3 with fault.

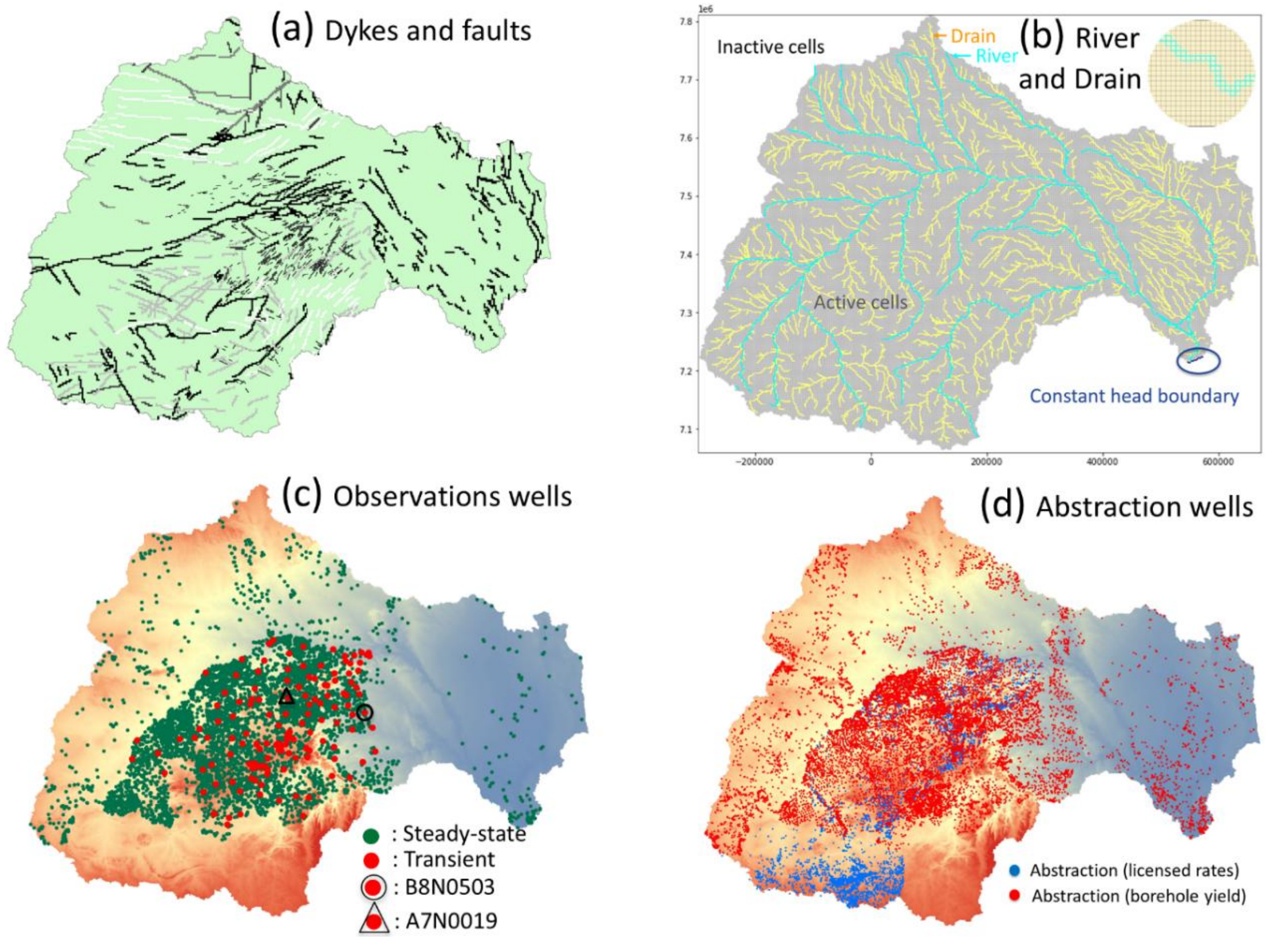

Figure S2. The distribution of dykes and faults (a); major rivers (aqua color) with their main distributaries and tributaries (yellow color) along with the model grid layout and boundary conditions (b); groundwater observation wells (c); and abstraction wells (d) in the LRB. The inset on the top-right corner of b shows the structured grid with a uniform resolution of 1x1 km.

ESM 2.5 WetSpass model development and tuning

WetSpass-M, which is the latest version of the WetSpass model and supports monthly calculations (Abdollahi et al., 2017), has been used to simulate monthly spatially distributed groundwater recharge. After collecting data from different sources (Table ESM2), spatially distributed raster maps of all model inputs (DEM, soil texture, slope, land cover, groundwater depth, and hydrometeorological data: rainfall, temperature, wind speed, and potential evapotranspiration (PET)) were prepared at a 1000 m resolution. Another input to the model is the number of rainy days. Details of the input data, including preparation techniques, are reported in Table ESM2. Prepared input maps and tables were then used to simulate spatially distributed groundwater recharge.

The model performance was evaluated in two steps using data from different sources: (i) available groundwater recharge from other studies in the LRB, and (ii) groundwater levels by coupling the model with the MODFLOW-2005 groundwater flow model. First, the simulated recharge was compared with the available groundwater recharge from other studies in the LRB (MacDonald et al., 2008; Mosase et al., 2019; Lindle et al., 2023). After that, the spatially distributed recharge obtained from the WetSpass-M model was used as an input for the MODFLOW model. The model was further calibrated and evaluated by coupling it with MODFLOW-2005 through minimizing the misfit between observed and simulated groundwater levels.

ESM 2.6 Model calibration and performance evaluation

Statistical indicators used for the model performance evaluation were: coefficient of determination (R^2), mean error (ME), mean absolute error (MAE), root mean square error (RMSE), relative root mean square error (rRMSE), Nash–Sutcliffe efficiency (NSE), Kling-Gupta efficiency (KGE, Gupta et al., 2009), Percent Bias (PBIAS) and residual quantile plot.

Values of relative root mean square error (rRMSE) closer to zero indicates better simulation capacity. The ranges of rRMSE >30%, 20 – 30%, 20 – 20%, and <10%, respectively, indicate poor, fair, good, and excellent simulation performance. The values of NSE vary from – α to +1, and it has no dimension. An NSE value close to 1 means better simulation efficiency. NSE values < 0.0, 0.0 – 0.35, 0.35 – 0.7, and > 0.7 indicate poor, fair, good and excellent simulation efficiency. The values of PBIAS close to zero mean better simulation capacity, whereas negative and positive values of PBIAS represent overestimation and underestimation bias, respectively. A KGE value of 1

indicates perfect agreement between simulations and observations, and a value of 0.6 or greater is generally considered to be satisfactory (Patil and Stieglitz, 2015). During model performance evaluation, more weight was given to KGE because of its robustness and high sensitivity.

ESM 2.7 Evaluation of management scenarios

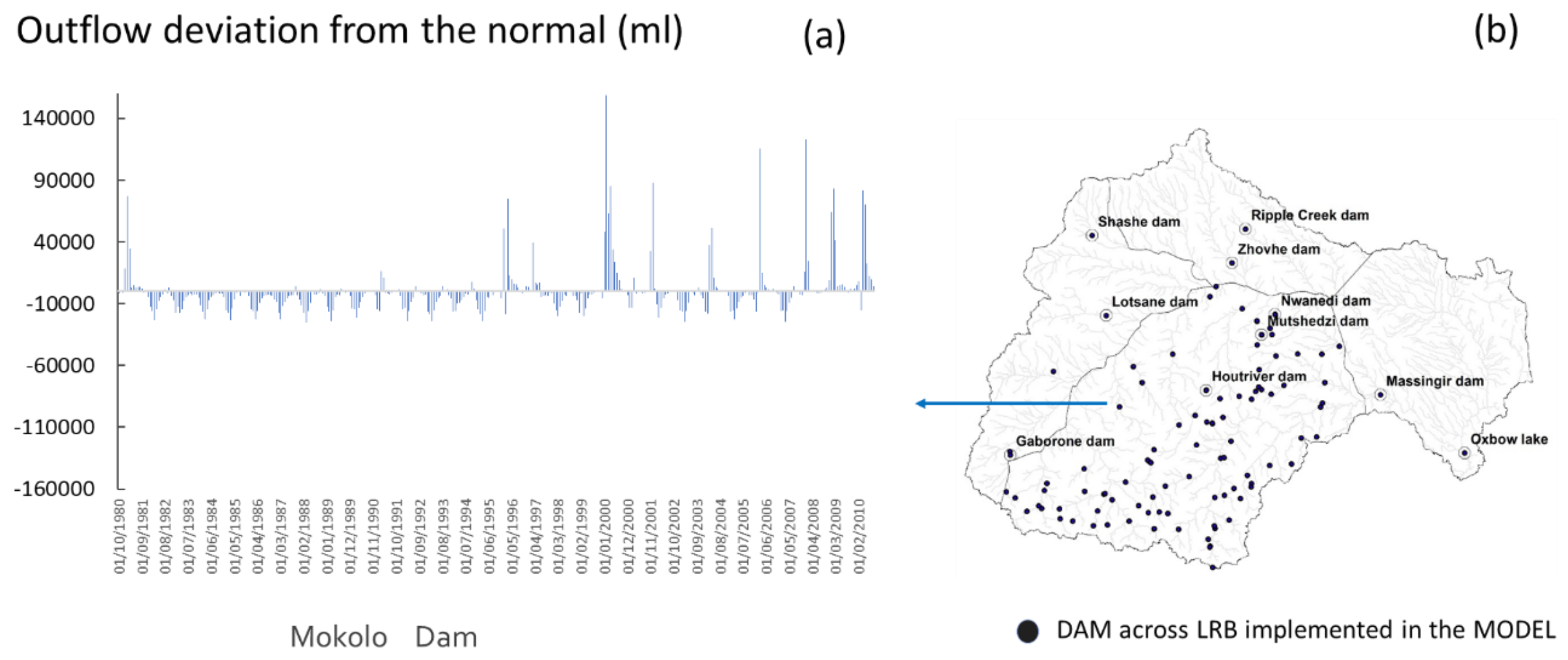

Figure S3. An example of outflow deviation from the normal (a) and the dam's location across the LRB (b).

Table S5. The weight fraction is calculated based on population density (Source: Table S2).

Population density 2020 (people per km^2)	Weight(s) fraction
0-1	0
1-2	0.1
2-5	0.2
5-10	0.3
10-20	0.4
20-30	0.5
30-40	0.6
40-50	0.7
50-100	0.8
>100	1.0

ESM 3.1 Workshops: identification and co-creation of management scenarios

In this study, an iterative MCGM approach has been proposed that involved stakeholders at every step of the modelling, incorporating their perspectives and expertise (see Section 2.2).

The discussion clearly highlighted that groundwater abstraction is increasing in the LRB to meet the increasing water demand from agricultural, domestic, and industrial water demand as a result of population growth, but also as a coping strategy to increasingly unreliable surface water sources as a result of increasing climate extremes. Additionally, it was mentioned that the frequency and intensity of hydrological extremes are rising in the LRB, posing a serious threat to livelihoods, as well as water and food security. Stakeholders also emphasized that deforestation is a significant concern in certain basin areas, while also noting the potential for afforestation in the LRB. However, the impact of both deforestation and afforestation on water availability and the hydrological system remains unclear, prompting their interest to explore this further. They also highlighted that MAR is currently being used in South Africa and they are interested in exploring the effect of expanding this measure. The same applies to sand dams in Zimbabwe and Botswana, large surface water reservoirs in Mozambique, and forecasting and early-warning systems. Stakeholders from Botswana and South Africa are more interested in local solutions, such as MAR using rainwater and runoff harvesting through local ponds and wells. They also want to explore the potential of MAR using injection wells. Meanwhile, stakeholders from Botswana and Zimbabwe prefer MAR using sand dams. Stakeholders emphasize the urgency of finding management policy recommendations to reduce the impacts of and increase resilience to hydrological extremes (floods and droughts), as well as to enhance groundwater sustainability throughout the basin.

During group discussions and transboundary workshops, model conceptualization (including the aquifer layering, basement weathering, fractures and dykes, ephemeral/permanent rivers, and groundwater abstraction (licensed and actual rate)) was iteratively improved based on stakeholder feedback and inputs.

Through iterative collaborative modelling and knowledge co-creation, seven management scenarios have been identified (section 2.5) aimed at achieving the following objectives: (i) reducing impacts and increasing resilience to hydrological extremes (floods and drought); (ii) enhancing groundwater sustainability throughout the basin; and (iii) understanding stakeholders' perception and preference on the effectiveness and feasibility of implementing measures for resilience and sustainability.

ESM 4.1 The impact of alternative model conceptualisations

To understand the sensitivity of the modeling choices made in this study – including the assumption of a uniform aquifer thickness, the 1000 m x 1000 m spatial resolution, and the representation of boundary conditions and structural controls – the impact of alternative model conceptualizations on groundwater levels and river-aquifer interaction uncertainty was evaluated. The reader is referred to the MSc thesis by de Vries, Wageningen University, 2025, for full results.

The impact of spatial resolution and river-aquifer interactions was assessed using an unstructured grid with higher resolution (100 m x 100 m) near rivers, compared to the original uniform 1000 m x 1000 m structured grid. The impact of structural controls and aquifer thickness was evaluated using three layering conceptualizations: (i) the original three-layer model, (ii) a four-layer model including an extra layer for alluvial sediments, and (iii) a five-layer model with a compacted bottom layer. The impact of boundary conditions was tested using alternative representations of (i) seasonal variability in river flow, (ii) groundwater inflow from the northwestern and southwestern boundaries (flow boundary), and (iii) the current no-flow boundary (excluding the sea-side).

Results show that both the direction and magnitude of groundwater-surface water interactions are strongly affected at the local scale by the model grid structure and the representation of river seasonality. However, groundwater head uncertainty stemming from the river grid structure remains limited to areas near the river. Alternative representations of geological stratification strongly affect groundwater head uncertainty, especially in regions with deep groundwater levels.

The results also demonstrate that while there are considerable impacts at the local scale, the broader basin-scale trends and river-aquifer interactions remain consistent with the original study and do not fundamentally alter the main findings. These additional analyses confirm the robustness of our results and the importance of considering multiple conceptual models to better understand structural modeling uncertainties for local-level applications.

References

Arvidsson, K., Stenberg, L., Chirindja, F., Dahlin, T., Owen, R., & Steinbruch, F. (2011). A hydrogeological study of the Nhandugue River, Mozambique–A major groundwater recharge zone. Physics and Chemistry of the Earth, Parts A/B/C, 36(14-15), 789-797.

Beasley, A.J. 1983. The hydrogeology of the area around Nyamandhlovu, Zimbabwe. Ph.D. Thesis. University of London.

Brassington, R. (1998). Field hydrogeology (second ed. ed). John Wiley & Sons.

Dippenaar, M. A., Witthüser, K. T., & Van Rooy, J. L. (2009). Groundwater occurrence in Basement aquifers in Limpopo Province, South Africa: model-setting-scenario approach. Environmental Earth Sciences, 59, 459-464.

Gupta, H. V., Kling, H., Yilmaz, K. K., & Martinez, G. F. (2009). Decomposition of the mean squared error and NSE performance criteria: Implications for improving hydrological modelling. Journal of hydrology, 377(1-2), 80-91.

Hengl, T., Mendes de Jesus, J., Heuvelink, G. B., Ruiperez Gonzalez, M., Kilibarda, M., Blagotić, A., ... & Kempen, B. (2017). SoilGrids250m: Global gridded soil information based on machine learning. PLoS one, 12(2), e0169748.

Holland, M., & Witthüser, K. T. (2011). Evaluation of geologic and geomorphologic influences on borehole productivity in crystalline bedrock aquifers of Limpopo Province, South Africa. Hydrogeology Journal, 19(5), 1065.

Hussey, S. W. (2003). The feasibility of sand-abstraction as a viable method of ground water abstraction (Doctoral dissertation, Loughborough University).

Larsen, F., Owen, R., Dahlin, T., Mangeya, P., & Barmen, G. (2002). A preliminary analysis of the groundwater recharge to the Karoo formations, mid-Zambezi basin, Zimbabwe. Physics and Chemistry of the Earth, Parts A/B/C, 27(11-22), 765-772.

Lekula, M., Lubczynski, M. W., & Shemang, E. M. (2018). Hydrogeological conceptual model of large and complex sedimentary aquifer systems–Central Kalahari Basin. Physics and Chemistry of the Earth, Parts A/B/C, 106, 47-62.

Lindle, J., Villholth, K. G., Ebrahim, G. Y., Sorensen, J. P. R., Taylor, R. G., & Jensen, K. H. (2023). Groundwater recharge influenced by ephemeral river flow and land use in the semiarid Limpopo Province of South Africa. Hydrogeology Journal, 31(8), 2291-2306

Mansell, M. G., & Hussey, S. W. (2005). An investigation of flows and losses within the alluvial sands of ephemeral rivers in Zimbabwe. Journal of Hydrology, 314(1-4), 192-203.

Marema, T. M., Molwalefhe, L., & Shemang, E. M. (2023). Characterisation of the Hydrogeological Properties of the Ntane Sandstone Aquifer Using Co-Seismic and Post-Seismic Groundwater Level Responses to the Mw 6.5 Moiyabana Earthquake, Central Botswana. Water, 15(10), 1947.

Masvopo, T. H. (2008). Evaluation of the groundwater potential of the Malala alluvial aquifer lower Mzingwane river Zimbabwe (Doctoral dissertation, University of Zimbabwe).

Maurice, L., Taylor, R. G., Tindimugaya, C., MacDonald, A. M., Johnson, P., Kaponda, A., ... & Gooddy, D. (2019). Characteristics of high-intensity groundwater abstractions from weathered crystalline bedrock aquifers in East Africa. Hydrogeology Journal, 27(2), 459-474.

Mulder, M. P. (1973). Water supply from river sand: Limpopo River at Messina. Department of Water Affairs and Forestry Report Gh3237, Pretoria, South Africa.

Nord, M. (1985). Sand Rivers of Botswana, Results from phase 2 of the Sand Rivers Project. Unpublished report, Department of Water Affairs, Government of Botswana, Gaborone.

Owen, R. J. S. (1989). The use of shallow alluvial aquifers for small scale irrigation with reference to Zimbabwe. Final report of ODA Project, 4239, 121.

Patil, S. D., & Stieglitz, M. (2015). Comparing spatial and temporal transferability of hydrological model parameters. Journal of Hydrology, 525, 409-417.

Snowy Mountains Engineering Corporation, EHES Consulting Engineers, 2006. National Water Master Plan Review. Volume 4 : Groundwater Resources. Department of Water Affairs (Unpublished report).

Thomas, E.G., & Hyde, L.W. (1972). Water storage in the Sand Rivers of Eastern Botswana with particular reference to storage on the Mahalapshwe River. Technical Note 33. United Nations Development Programme (UNDP). Government of Botswana, Gaborone.

United Nations. 1989. Groundwater in Eastern, Central and Southern Africa: Botswana. In: Groundwater in Eastern, Central and Southern Africa. Natural Resources/Water Series No.19, ST/TCD/6. ISBN 92-1-104223-2.

Upton, K., O Dochartaigh, B., & MacDonald, A. (2017). Africa Groundwater Atlas: Making African groundwater information more visible and accessible. International Conference on Geology, Mining, Mineral and Groundwater Resources, Livingstone, Zambia, 11-13 July 2017.

Wekesa, S. S., Stigter, T. Y., Olang, L. O., Oloo, F., Fouchy, K., & McClain, M. E. (2020). Water flow behavior and storage potential of the semi-arid ephemeral river system in the Mara Basin of Kenya. Frontiers in Environmental Science, 8, 95.
